# Chemical Scissors Tailored Nano-Tellurium with High-Entropy Morphology for Efficient Foam-Hydrogel-Based Solar Photothermal Evaporators

**DOI:** 10.1007/s40820-023-01242-y

**Published:** 2023-12-08

**Authors:** Chenyang Xing, Zihao Li, Ziao Wang, Shaohui Zhang, Zhongjian Xie, Xi Zhu, Zhengchun Peng

**Affiliations:** 1 State Key Laboratory of Radio Frequency Heterogeneous Integration, Shenzhen University, Shenzhen, 518060 People’s Republic of China; 2 Center for Stretchable Electronics and NanoSensors, College of Physics and Optoelectronic Engineering, Shenzhen University, Shenzhen, 518060 People’s Republic of China; 3 School of Science and Engineering, The Chinese University of Hong Kong, Shenzhen, Shenzhen, 518172 People’s Republic of China; 4 Institute of Pediatrics, Shenzhen Children’s Hospital, Shenzhen, 518038 Guangdong People’s Republic of China; 5 International Collaborative Laboratory of 2D Materials for Optoelectronics Science and Technology of Ministry of Education, Institute of Microscale Optoelectronics, Shenzhen University, Shenzhen, 518060 People’s Republic of China

**Keywords:** Tellurium, High entropy, Electrochemical modification, Solar absorption, Evaporation rate

## Abstract

The development of tellurium (Te)-based semiconductor nanomaterials for efficient light-to-heat conversion may offer an effective means of harvesting sunlight to address global energy concerns. However, the nanosized Te (nano-Te) materials reported to date suffer from a series of drawbacks, including limited light absorption and a lack of surface structures. Herein, we report the preparation of nano-Te by electrochemical exfoliation using an electrolyzable room-temperature ionic liquid. Anions, cations, and their corresponding electrolytic products acting as chemical scissors can precisely intercalate and functionalize bulk Te. The resulting nano-Te has high morphological entropy, rich surface functional groups, and broad light absorption. We also constructed foam hydrogels based on poly (vinyl alcohol)/nano-Te, which achieved an evaporation rate and energy efficiency of 4.11 kg m^−2^ h^−1^ and 128%, respectively, under 1 sun irradiation. Furthermore, the evaporation rate was maintained in the range 2.5–3.0 kg m^−2^ h^−1^ outdoors under 0.5–1.0 sun, providing highly efficient evaporation under low light conditions.

**Supplementary Information:**

The online version contains supplementary material available at 10.1007/s40820-023-01242-y.

## Highlights

- Precise exfoliation and modification of nano-Tellurium was realized by adopting a room-temperature ionic liquid as the electrolyte.
- Nano-Tellurium with high-entropy morphology can offer greater solar absorption and more kinds of surface chemical groups, giving rise to superior photothermal properties.
- Nano-Tellurium-poly(vinyl alcohol)-based photothermal foam hydrogels, with high compressibility, excellent water transport rate and low evaporation enthalpy of water, combined with a new heat-supply model, achieve an evaporation rate of 4.11 kg m^−2^ h^−1^ with energy efficiencies up to 128% under 1 sun irradiation, which are the highest values for semiconductor-based nanocomposites reported so far.

**Supplementary Information:**

The online version contains supplementary material available at 10.1007/s40820-023-01242-y.

## Graphical abstract

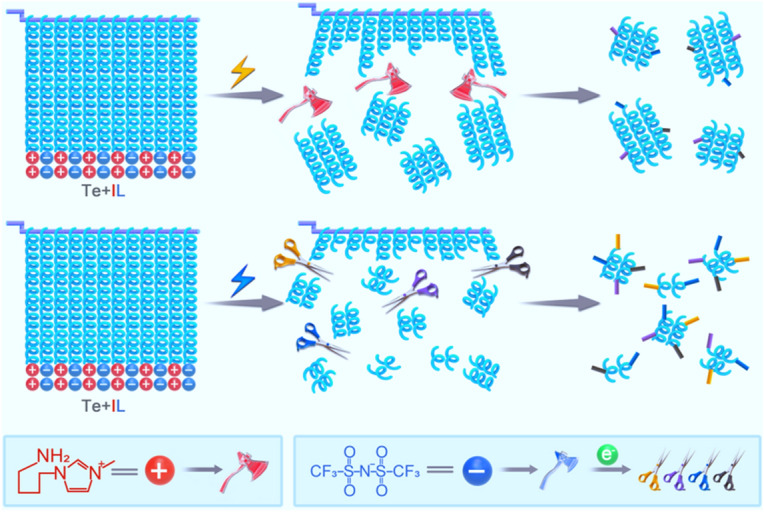

## Introduction

The mono-elemental group-VIA semiconductor tellurium (Te) has drawn extensive research attention owing to its potential application in optoelectronic, photothermal, and thermoelectric devices [1–6]. Te has a narrow energy bandgap (*E*_g_) of 0.30 eV in its bulk state. Te-based nanomaterials (nano-Te) show layer-dependent *E*_g_ values [7], similar to the group-VA element black phosphorus (BP) [8–12]. Nano-Te also exhibits both plasmonic- and Mie-type resonances [13], together with low thermal conductivity [14–16], making it a promising material for solar photothermal applications that use solar radiation over the wavelength range 250–2500 nm [17]. Recent strategies to synthesize nano-Te include chemical methods such as common redox reactions [18, 19] and hydrothermal reactions [20, 21]; biosynthesis [22, 23]; and physical methods such as liquid phase exfoliation (LPE) [24–26], physical vapor deposition (PVD) [27], and laser ablation [13]. However, nano-Te fabricated by these methods exhibits unsatisfactory solar absorption, particularly in the infrared (IR) region. This feature is partly attributed to the regular nature of Te nanostructures, i.e., zero-dimensional (0D) quantum dots (QDs) [28]; one-dimensional (1D) nanowires [29] or nanoneedles [30]; and two-dimensional (2D) nanosheets [24, 27].

Ideal solar photothermal agents (SPAs) have two main requirements: They should effectively absorb solar radiation, and they should have a high light-to-heat conversion efficiency. The former quality is closely related to the electronic structure and morphology of the SPA [31, 32], and the latter relates to its surface state, including defects, impurities, and surface termination [33]. Accordingly, for nano-Te to fulfill its potential as a solar photothermal agent, it should have multi-dimensional and diverse structures with a high specific surface area to absorb solar energy over a broad range along with an abundance of surface defects and/or functional groups to transform photoexcited electrons into heat energy. Accordingly, we aimed to develop a nano-Te material system for SPAs.

Electrochemical exfoliation (ECE) strategies have been applied for the fast and effective preparation of few-layer 2D inorganic materials, particularly for layered materials such as classic graphene and BP [34, 35]. Typically, electrolytes and solvents play an important role in intercalating and decorating the target objects [9, 36]. Recently, room-temperature ionic liquids (ILs), composed only of cations and anions, have been applied as intercalation agents to produce graphene [37–39], graphene QDs [40], BP QDs [41], and BP nanosheets [42]. Tang et al. reported the use of a fluoride-containing IL to exfoliate BP crystals in an electric field [41, 42]. In this strategy, the BF_4_^−^ anions decomposed into F^−^, which chemically bind with the BP surface. Consequently, both air stability and light absorption of the IL-modified BP were improved.

Both the cations and anions in ILs can be electrolyzed in an electric field [43–49]. For instance, IL imidazolium cations can be electrolyzed into imidazolium radicals [44] and even alkyl radicals [50]. Interestingly, the bis[(trifluoromethyl) sulfonyl]imide (NTf_2_) anions can be electrolyzed into several active species [43], including the radical ∙NSO_2_CF_3_‾ and the anions SO_2_CF_3_‾, NSO_2_‾, SO_2_‾, CF_3_‾, and even F ‾. By these means, metal fluorides can be formed on electrodes [51–53]. Based on these previous findings, these chemical species have the potential to act as electrolytes and participate in exfoliation procedures. Furthermore, for newly exfoliated nanomaterials, where physical interactions between sheets or chains have been broken, there are transiently and highly reactive surfaces. If active electrolyzed species are present in the ECE system, new nanomaterials may be readily grafted to them, giving rise to surface-decorated Te nanomaterials if bulk Te is used as a working electrode. Such an unique preparation of Te-based nanomaterials by ECE of Te has not been reported previously [54]. Therefore, an ECE strategy featuring electrolyzable ILs as electrolytes may be expected to provide functional nano-Te from bulk Te under electrochemical conditions.

In this work, for the first time, we report the electrochemical synthesis of nano-Te with the use of an electrolyzable ionic liquid [1-aminopropyl-6-methylimidazolium]{[bis(trifluoromethyl)sulfonyl]imide}, [C_3_NH_2_MIm][NTf_2_], as the electrolyte. By adjusting the applied voltage, precise exfoliation of Te by IL anions and/or cations realizes a wide variety of Te morphologies owing to the different exfoliation mechanisms of the IL components. Anion-induced nano-Te has a varied morphology, which increases solar absorption, and provides tunable water-stability for at least 14 days in water. Accordingly, this form of nano-Te was used as a photothermal nanofiller in poly (vinyl alcohol) (PVA)-based foam hydrogels generated by a foam phase conversion strategy. These PVA/nano-Te foam hydrogels combine the advantages of a foam (high porosity, high water transport, excellent solar absorption, and photothermal efficiency) and a hydrogel (low water evaporation enthalpy). Thus, excellent evaporation performances under 1 sun (indoor) and weak light irradiation (outdoor) conditions were achieved.

## Experimental and Calculation

### Fabrication Strategies

#### Electrochemical Strategy

The nano-Te materials were fabricated using a classic two-electrode electrochemical method with an electrolyzable ionic liquid [1-aminopropyl-6-methylimidazolium] {[bis (trifluoromethyl) sulfonyl] imide}, [C_3_NH_2_MIm][NTf_2_], as the electrolyte (0.5 M). In brief, commercial bulk Te crystal was used as working electrode while Pt as the counter one, and acetonitrile (CH_3_CN) as the solvent. Bias potential, including positive + 1, + 2, + 3, + 4, and + 5 V, and negative − 1, − 2, − 3, − 4, and − 5 V, was applied onto Te electrode. After a certain time of exfoliation, the nano-Te/CH_3_CN colloidal solution was obtained, followed by centrifuged at 200 rpm for 10 min to remove unexfoliated bulk Te, filtration, washing with CH_3_CN, methanol, and water. The final solid nano-Te can be obtained by freeze-drying operation.

#### Fabrication of PVA-Based Hydrogels and Foam Hydrogels

*PVA Hydrogel* Typically, PVA solution, glutaraldehyde (GA), HCl solution, and deionized (DI) water were fully mixed at 0 °C (with a moderate stirring rate of 500 rpm), followed by a heating process at 40 °C for 10 min to get gelation. The final PVA hydrogels were obtained by fully dialyzing them with water at room temperature.

*PVA Foam Hydrogel* Differed from the above method of PVA hydrogel, the PVA foam hydrogel precursor was first fully foamed by using a high stirring rate of 35,000 rpm at 0 °C after its fully mixing under low stirring speed. Then, a similar heating cross-linking strategy was used to stabilize the foamy system, followed by being frozen at − 20 °C for 1 h and thawed at room temperature. The final PVA foam hydrogels were obtained by repeatedly squeezing them in hot water to replace the bubble-introduced air with water.

*PVA/F-127 Foam Hydrogel* To further homogenize and stabilize the bubbles in PVA system, nonionic surfactant PEO_106_-PPO_70_-PEO_106_ (commercial name: Pluronic F-127) was used and its solution with a 100 mg mL^−1^ concentration was used to replace the above DI water. Similar procedures can obtain the PVA/F-127 foam hydrogels.

*PVA/F-127/Nano-Te Foam Hydrogel* The as-prepared nano-Te was introduced into the PVA/F-127 system to act as solar absorbent and photothermal nanofiller. Prior to foaming, the nano-Te was thoroughly mixed with PVA/F-127 system to get a homogeneous state. PVA/F-127/nano-Te foam hydrogels with various nano-Te concentrations can be obtained according to the above preparation strategy.

### Characterization

*Nano-Te* The morphology was characterized by using transmission electron microscopy (TEM) with a type of Tecnai G2, Spirit120kV. The surface termination was detected by X-ray photoelectron spectroscopy (XPS) and Fourier-transform infrared spectroscopy (FTIR), respectively. The crystalline behaviors were recorded by X-ray diffraction (XRD), and the vibration behaviors were analyzed by Raman spectra. The solid solar absorption property was measured by using an instrument from 200 to 2500 nm.

*Foam Hydrogels* The cross-section morphology was observed by using scanning electron microscopy (SEM). Before testing, a thin of Pt layer was sprayed onto the surface of samples to improve their conductivity. The porous property of samples was evaluated by recording their pore size distribution and porosity by using mercury intrusion method. The water transport and retain property were tested by testing water absorption rate within certain time after absorbing water and water mass after centrifugation after 2500 rpm for 10 min, respectively. The solar absorption behaviors were calculated by deducting the transmittance and reflections of samples at seawater wet and dry forms. The water evaporation enthalpy was measured by calculating the mass loss of water in samples under certain conditions that has dark conditions. The mechanical properties were evaluated by testing their compressibility as a frequency of 2 s^−1^ at a fixed compressive ratio of 85%.

*Indoor and Outdoor Solar Desalination* Foam hydrogels were inserted into the white heat-insulative foam or black heat-absorption foam, namely thermal isolation model and heat-supply model, respectively, to construct the solar evaporators. Owing to their self-floating property of both foam hydrogels and foams, these evaporators were floating onto the seawater surfaces. The mass and surface temperature changes of the systems were recorded by using electron balances and infrared thermal imager during evaporation procedure at room temperature. The simulated light was fixed at one sun by calibrated by using an optical power meter before use. The evaporation rate (*ν*) and energy efficiency (*η*) were calculated by using the following equations. At least three samples were used to report the average value. The real seawater was obtained Shenzhen Bay, Shenzhen, China, and was used after a simple filtration to remove any of visible floating objects.

In outdoor, the large-scale solar evaporators with same volume ratios as the one indoor were evaluated from 8:00 am to 18:00 pm every day during period from September 4, 2022, to October 6, 2022. At every critical time data, the *ν*, surface temperature, ambient temperature, and solar light intensity were recorded. For instance, at 8:00 am, the *ν* indicates the average value between the time 7:00 am and 8:00 am. Another sample, at 9:00 am, the *ν* indicates the average value between the time 8:00 am and 9:00 am. Except the rainy day and partly of rainy day at one day, 32 days and 341 data of every time point were continuously detected.

All calculations based on density functional theory (DFT) were utilized through the Vienna ab initio simulation package (VASP) [55]. Interactions arising from exchange correlation were depicted through the generalized gradient approximation (GGA) configured by the Perdew–Burke–Ernzerhof (PBE) approach [56]. The DFT-D3 scheme facilitated the inclusion of van der Waals (vdW) interactions [57, 58]. The energy threshold of the plane wave base was defined at 500 eV, with a 3 × 3 × 1 k point grid sampled through the Monkhorst–Pack technique ensuring sufficient precision for Brillouin zone integration [59]. The conjugate gradient (CG) algorithm enabled complete relaxation of all structures until a force less than 0.005 eV/Å and an energy fluctuation under 10–8 eV were achieved. To emulate the surface of Te, we adopted a 2 × 2 × 1 monolayer supercell and sliced the crystal along the (0 0 1) orientation. The vacuum domain in the z-direction was prescribed at 15 Å to preclude inter-layer interactions. Different vibrational modes of adsorbate groups were simulated by changing corresponding bond length.

The adsorption energies (*E*_ads_) were calculated by:

1 $${E}_{\mathrm{ads}}={E}_{\mathrm{ad}}+{E}_{\mathrm{slab}}-{E}_{\mathrm{ad}+\mathrm{slab}}$$

where *E*_ad_, *E*_slab_, and *E*_ad+slab_ represent the total energy of the adsorbate, the relaxed slab, and the slab adsorbed by the adsorbates, respectively.

## Results and Discussion

### Entropy and the Photothermal Effect

The temperature of this system can be modeled with a two-slab model as reported by Gartner [60], in which the temperature distribution between two infinite semiconductor slabs is calculated. One of the slabs is illuminated and heat is transferred between the slabs. The temperature at the illuminated slab is ${T}_{1}$, and the temperature at distance $w$ is denoted as ${T}_{2}$. The solution for ${T}_{1}$ is given as:

2 $${\mathrm{T}}_{1}=-\left\{\mathrm{\alpha }{\mathrm{T}}_{0}-\frac{\upkappa }{\mathrm{w}}{\mathrm{T}}_{2}+{\mathrm{f}}_{\mathrm{AC}}\left[1-\frac{\mathrm{L}}{\mathrm{\tau w}}\times \frac{2\mathrm{D}-\left(\mathrm{D}-{\mathrm{s}}_{2}\right){\mathrm{e}}^{-\mathrm{W}}-\left(\mathrm{D}+{\mathrm{s}}_{2}\right){\mathrm{e}}^{\mathrm{W}}}{\left(\mathrm{D}+{\mathrm{s}}_{1}\right)\left(\mathrm{D}+{\mathrm{s}}_{2}\right){\mathrm{e}}^{\mathrm{W}}-\left(\mathrm{D}-{\mathrm{s}}_{1}\right)\left(\mathrm{D}-{\mathrm{s}}_{2}\right){\mathrm{e}}^{-\mathrm{W}}}\right]\right\}\times {\left(\frac{\upkappa }{\mathrm{w}}-\mathrm{\alpha }\right)}^{-1}$$

where $\alpha$ is a constant for the heat emitted via radiation; $\kappa$ is the thermal conductivity of the material; ${f}_{AC}$ is the radiated flux absorbed exclusively under carrier generation in the bulk or on the surface of the sample; $D$ is the ambipolar diffusion constant given by $(n+p)/(n/{D}_{p}+p/{D}_{n})$ with $n, p, {D}_{n}, {D}_{p}$ relating to the concentrations and diffusion constants of the electrons and holes; $L$ is the diffusion length of excess pairs as $\sqrt{D{\tau }_{p}}$ with ${\tau }_{p}$ as the lifetime of holes; and $\mathcal{D}=D/L$, $W=w/L$, ${s}_{1},{s}_{2}$ are the recombination velocities of the illuminated slab and the other slab. The shape of this function is determined by the reciprocal terms on ${T}_{2}$ and on the outside, which have a singularity at $w=\kappa /\alpha$. Thus, when using this theory, the range where $w$ approaches this singularity is invalid.

We now consider the size distribution effect, i.e., the entropy effect. Suppose that the distribution is identical for $N$ particles of the same $w={w}_{0}$, the ${T}_{1}$ value of this distribution is:

3 $${T}_{1}^{\mathrm{identical}}=\sum_{i=1,\dots ,N}{T}_{1}({w}_{0})\frac{1}{N}={T}_{1}({w}_{0})$$

Assuming that the distribution is Gaussian with mean ${w}_{0}$ and standard deviation $\sigma$, the ${T}_{1}$ value is described by a Gaussian function as:

4 $${T}_{1}^{\mathrm{Gaussian}}={\int }_{{w}_{t}=0}^{{w}_{t}=\infty }{T}_{1}\left({w}_{t}\right)\frac{1}{\sqrt{2\pi {\sigma }^{2}}}{e}^{-\frac{1}{2}{\left(\frac{{w}_{t}-{w}_{0}}{\sigma }\right)}^{2}}d{w}_{t}\approx \frac{{T}_{2}\kappa \left(2{\alpha }^{2}{\sigma }^{2}+5{\left(\kappa -\alpha {w}_{0}\right)}^{2}\right)-{T}_{0}\alpha \left(5{\kappa }^{2}{w}_{0}+5{\alpha }^{2}{w}_{0}^{3}+2\alpha \kappa \left({\sigma }^{2}-5{w}_{0}^{2}\right)\right)}{3\sqrt{2\pi }{\left(\kappa -\alpha {w}_{0}\right)}^{3}}$$

According to the numerical values in Gartner’s model [60] and other publications [61–63], the numerical parameters may be taken as *S*_1_ = 100 cm s^−1^, *S*_2_ = 100 cm s^−1^, *T*_1_ = 298 K, *T*_2_ = 358 K, $\mathcal{D}$ = 50 cm^2^ ν^−1^ s^−1^, *L* = 1 cm, *τ* = 0.02 s, *f*_*ac*_ = 0.01 W cm^−2^, *κ* = 0.5 W K^−1^, *α* = 2 W K^−1^, *σ* = 0.001 cm, and *w*_0_ = 0.002 cm. Hence, the entropy effect can be calculated as shown in Fig. S1. The Gaussian distribution significantly increases the temperature ${T}_{1}$, which means that high-entropy semiconductors are likely to have greater photothermal effects.

### Electrochemical Exfoliation of Te

**Fig. 1:**
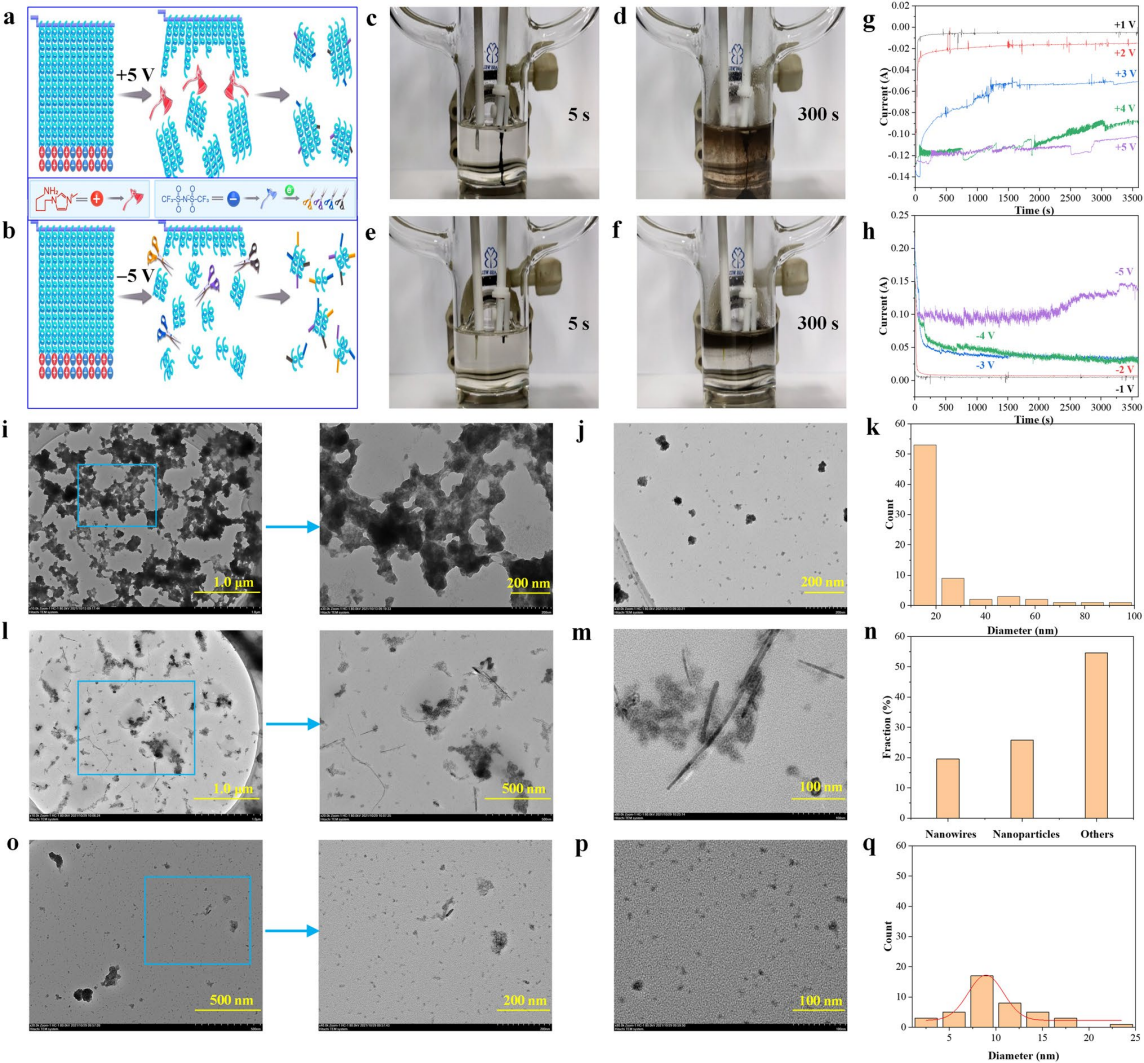
IL-assisted precise electrochemical synthesis of nano-Te. **a, b** Schematic of the intercalation and exfoliation of bulk Te by IL ions and their corresponding electrolyzed active species under opposite polarities. A typical standard two-electrode setup, including a Te crystal working electrode and high-purity Pt counter electrode for electrochemical modification of **c, d** Te under + 5 V or **e, f** − 5 V bias potentials**. g, h** Exfoliation current versus time in the preparation of nano-Te under various applied voltages. Typical TEM images and size distribution of nano-Te obtained from Te electrodes **i–k** at + 5 V and **l–q** at − 5 V

To fabricate high-entropy Te semiconductors, a common electrochemical exfoliation strategy was used. A bulk Te crystal was selected as a working electrode and fixed onto a Pt clamp, and another Pt sheet was used as a counter electrode (Fig. S2). As illustrated in Fig. 1a, b, bulk Te exists as chain-like structures with weak van der Waals interactions between chains allowing them to be broken by external force. The IL is composed of anions and cations, which can both interact with the Te chains under an electric field in the electrolyte. Therefore, by adjusting the electric field direction, cations or anions from the IL can be selectively interacted with the bulk Te. Note that the IL used here, [C_3_NH_2_MIm][NTf_2_], can be electrolyzed or decomposed into several active species, including radicals and anions. Hence, there are many modes of physical exfoliation and chemical bonding with Te.

At an applied potential of + 5 V to the Te electrode (Fig. S3) (Movie S1), exfoliation of Te is observed within approximately 2 s, while bubbles appear at the surface of the Pt counter electrode, which were determined not to originate from the electrolysis of water or CH_3_CN(not shown here). At 5 s (Fig. 1c), filament-like black products are clearly observed at the Te electrode. The expanded volume of Te indicates successful intercalation of the IL. Prolonged exfoliation improves the yield of this product (labeled nano-Te @ + 5 V) (Fig. 1d), which finally presents as a deep black colloidal solution in CH_3_CN (Fig. S3). Notably, when the electric field polarity at the Te electrode is changed (i.e., − 5 V, Fig. S3) (Movie S2), several distinct phenomena occur. First, the Te electrode exhibits slow exfoliation (from approximately 5 s) (Fig. 1e) and the exfoliated products float on the electrolyte surface (from approximately 15 s) (Fig. S3).

Finally, a clear two-phase interface is formed (at approximately 300 s) (Fig. 1f). A prolonged reaction time also increases the yield. In this case, no bubbles are observed at the Pt counter electrode, instead appearing at the Te working electrode. Similarly, the as-prepared Te products, denoted nano-Te @ − 5 V, appear black in CH_3_CN (Fig. S3). However, the corresponding filtered solutions of the above two products have different colors (Fig. S4). In addition, the exfoliation of bulk Te, under either positive or negative bias, had a threshold potential at approximately + 3 and − 3 V, as shown in Figs. 1g, h and S5, respectively. On exceeding these threshold potential values, the absolute values of exfoliation current start to increase sharply with applied potential. The chemical stability of the as-prepared nano-Te @ − 5 V in water was evaluated simply by observing its color change. As shown in Fig. S6, the bare nano-Te@ − 5 V shows moderate stability for 7 days before showing clear degradation at 14 days. In contrast, glutathione (GSH)-modified nano-Te exhibits stability in water for 14 days, indicating that the stability of nano-Te@ − 5 V may be modified with the use of appropriate polymers.

### Morphologies of Nano-Te

The microstructures of the nano-Te samples were evaluated by TEM imaging, as shown in Fig. 1i–q. The nano-Te @ + 5 V shows porous and continuous structures (Fig. 1i). The pore sizes are the range of 20–300 nm with irregular morphologies (Fig. 1i). The high-magnification image in Fig. 1j also shows the presence of 0D nanoparticles with diameters of 10–30 nm (Fig. 1k). Conversely, nano-Te @ − 5 V shows a wide variety of different architectures, as shown in Fig. 1l, m. 1D nanofibers (20%) with lengths of 100 nm to 1.0 μm, 0D nanoparticles (26%), and other nanostructures (54%) are observed (Fig. 1n). In addition, smaller 0D nanoparticles (Fig. 1o) and even quantum dots (QDs) (Fig. 1p, q) can also be observed. Exfoliation of nano-Te at + 5 V is rapid and produces particles with relatively large dimensions. The macroscopic filament-like appearance (Fig. 1c) is reflected by a continuous microscopic structure (Fig. 1i). However, in the case of the sample prepared at − 5 V, the exfoliation proceeds slowly such that small nano-Te microstructures are formed, resulting in the biphasic mixture observed (Fig. 1f). Owing to the chain-like structure of Te, the nano-Te materials feature continuous structures for the + 5 V sample and 1D nanofibers for the − 5 V sample. The nano-Te @ − 5 V sample shows a more diverse variety of morphologies, i.e., high-entropy morphology, which may be beneficial for its application to solar desalination.

### Physical Properties of Nano-Te

**Fig. 2:**
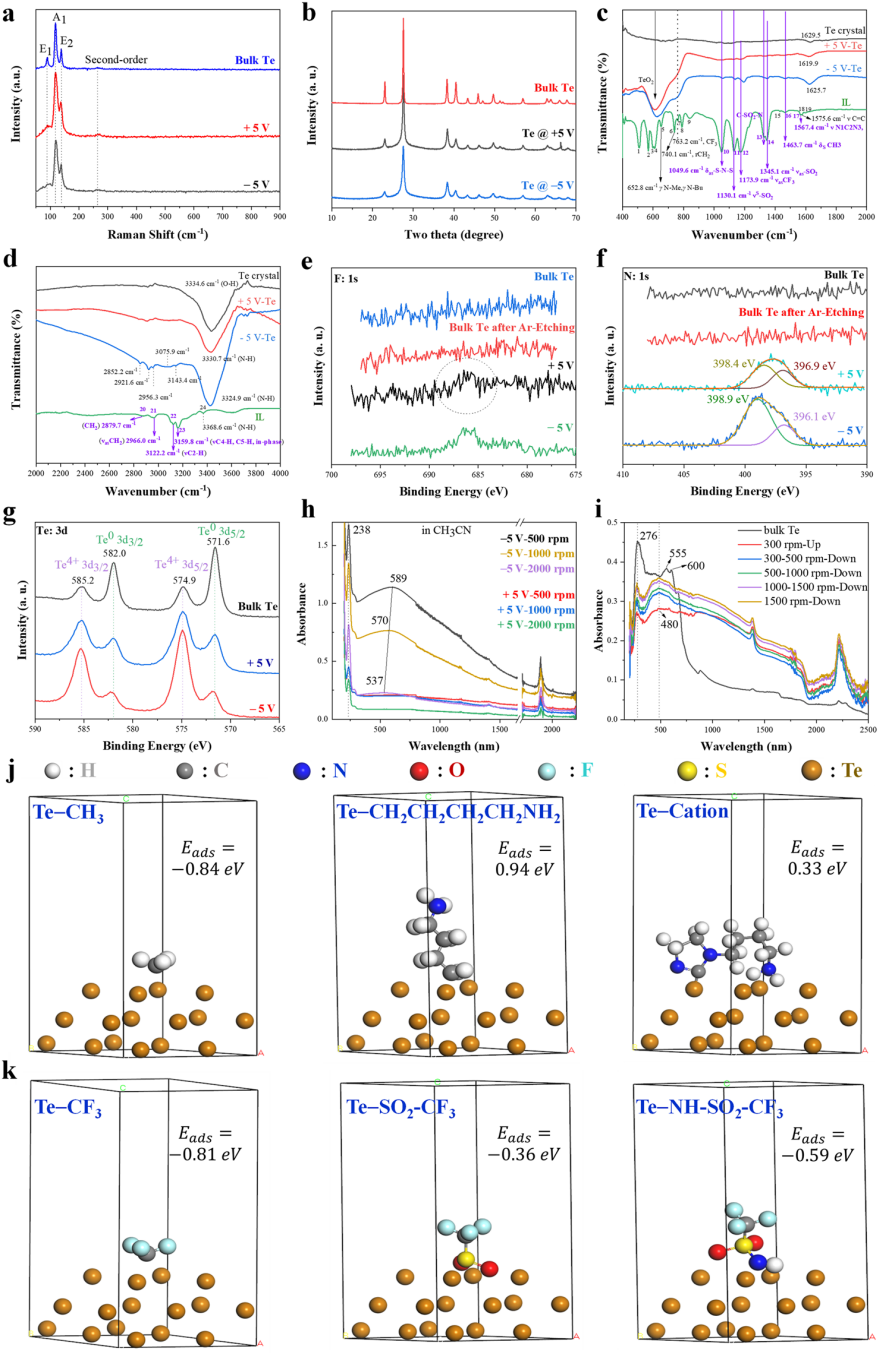
Physical properties of bulk Te and nano-Te samples under different conditions. **a** Raman spectra; **b** XRD patterns; **c**–**d** FTIR spectra; **e–g** XPS for **e** F, **f** N and **g** Te elements. Light absorption spectra of **h** nano-Te samples in CH_3_CN and **i** a solid nano-Te @ − 5 V sample over the solar spectral region. **j, k** Atomic structures and the corresponding adsorption energy of specific functional groups terminated Te at 001 face

Relative to bulk Te, nano-Te obtained at + 5 or − 5 V features similar Raman characteristics (Fig. 2a) and crystalline properties (Fig. 2b). No crystalline TiO_x_ is observed in these measurements [64–66]. However, TiO_x_ is detected by FTIR and XPS characterizations. For instance, bulk Te exhibits weak absorption (Fig. 2c) and clear high-valence Te-element signals (Fig. 2g), indicating a trace amount of TiO_x_ on its surface, probably because of oxidation in air. Interestingly, such TiO_x_ could absorb water molecules from the air to produce a strong hydrogen-bind interaction, as reflected by the strong absorption at 3334.6 cm^−1^ (Fig. 2d). For the nano-Te samples, apart from the strong Te–O bond, other weak functional groups are observed (Fig. 2c). In its pure form, the IL used exhibits absorption peaks at 763.2 cm^−1^ (CF_3_) [67], 1049.6 cm^−1^ (δ_as_-S–N–S) [67–69], 1130.1 cm^−1^ (ν^S^-SO_2_) [68, 69], 1173.9 cm^−1^ (ν_as_-CF_3_) [43], 1345.1 cm^−1^ (ν_as_-SO_2_) [67], and similar peaks are observed in the nano-Te spectra, in particular for nano-Te @ − 5 V. The characteristic absorptions for the imidazolium ring at 1567.4 cm^−1^ (ν N1C2N3) [68] and 1575.6 cm^−1^ (ν C = C) [70] are not observed in the nano-Te samples (Fig. 2c). However, clear alkyl C–H absorption peaks are observed at 1463.7 cm^−1^ (δ_s_ CH_3_) (Fig. 2c), 2852.2, 2921.6, and 2956.3 cm^−1^ in Fig. 2d, which are ascribed to CH_2_ vibration [67–69, 71] when compared with that of the pure IL used (Fig. 2d), suggesting that alkyl side chains instead of imidazolium ring may graft onto the nano-Te surfaces. These FTIR results indicate that the two types of nano-Te are both terminated by the fragmented chemical groups originating from the electrolyzed anions in the IL. Furthermore, nano-Te @ − 5 V appears to bear more functional groups in view of the presence of relatively strong peaks.

This surface chemical modification was further confirmed by the XPS measurements, as shown in Fig. 2e–g. Both nano-Te samples contain some F, and these peaks are relatively intense for nano-Te @ − 5 V compared with those for nano-Te @ + 5 V (Fig. 2e). Signals from N are also observed in a broad shoulder peak for both nano-Te samples (Fig. 2f). The above F and N signals are not found for bulk Te, indicating they originate from the IL. Additionally, the broad N peak can be deconvoluted into two possible peaks, which may be assigned to N from the anion and N from amino groups in the side chains from the imidazolium ring, considering that the N from imidazolium cations has a higher binding energy (> 400 eV) relative to that of anions [72–74]. Furthermore, both nano-Te samples show the presence of S at a lower binding energy (Fig. S7). Bulk Te also contains some S, which may be attributed to S doped into the commercial bulk Te. The above XPS results suggest that both nano-Te samples contain F-, N-, and S-containing chemical groups, which is in good agreement with the FTIR results.

The UV–Vis–NIR absorption spectra of the nano-Te samples recorded in CH_3_CN are shown in Fig. 2h. In the case of nano-Te @ − 5 V obtained after 500 rpm centrifugation, two distinct absorption peaks at 238 and 589 nm are observed, which can be attributed to the allowed direct transition from the valence band (VB) to the conduction band (CB) and an indirect forbidden transition, respectively. Furthermore, on elevating the centrifugation speed to 1000 and 2000 rpm, the first direct VB → CB transition absorption remains unchanged; however, the second forbidden indirect transition absorption is blue-shifted and has lower intensity. These unique absorption behaviors can be attributed to the diverse morphologies and dimensions of nano-Te @ − 5 V, which has high-entropy morphology. Therefore, relative to the samples obtained at high centrifugation speeds, those obtained at lower centrifugation speeds are more diverse in morphology, which provides better and broader optical absorption in the visible-light region. Regrettably, the nano-Te @ + 5 V sample, which has larger continuous microstructures, exhibits lower absorption of visible-IR light. Figure 2i shows the light absorption behaviors of the solid nano-Te @ − 5 V samples from different fractional centrifugation components. The solid bulk Te also presents two absorption features, a sharp peak at 276 nm and another broad peak in the range 555–600 nm, assigned to the two aforementioned transitions. However, bulk Te has poor optical absorption in the region 600–2500 nm. Nano-Te @ − 5 V samples from different fractional centrifugation components show broad absorption. Thus, nano-Te @ − 5 V has a broader solar absorption capacity than that of bulk Te. A combination of samples from centrifugation portions obtained in the range 300–1500 rpm (Fig. 2i) shows excellent optical absorption similar to that for the sample obtained at 500 rpm (Fig. 2h).

### Mechanism of Exfoliation and Functionalization of Te

Previous studies have revealed that ILs can be electrolyzed at potentials over their electrochemical windows [42–48]. Furthermore, NTf_2_ anions can be reductively and successively decomposed into several active species, such as ∙NSO_2_CF_3_‾, SO_2_CF_3_‾, NSO_2_CF_3_‾, SO_2_‾, and CF_3_‾ [43, 48], and can also be electrooxidized into NTf_2_∙ [75, 76]. The gaseous product CHF_3_ indicates reductive degradation of NTf_2_ anions. Furthermore, fluoride is also detected on the surfaces of the electrodes (LiF and MgF_2_) [51–53]. Imidazolium cations can be reductively transformed into dimers and alkyl radicals [50, 77], and these products are more colored in solution relative to those from NTf_2_ anions [78]. The density functional theory (DFT) calculation for the adsorption energies (*E*_ads_) of the aforementioned specific chemical functional groups onto the Te surface is shown in Fig. 2i, k, respectively. The − CH_3_ (*E*_ads_= 0.84 eV) and − NH_2_(*E*_ads_= 0.71 eV) groups, the partial side chains of the imidazolium cations, were found to have a potential to chemically bond with Te due to their negative E_ads_ values. However, the whole imidazolium cation ring with an *E*_ads_ value of 0.33 eV cannot graft onto the Te. On the other word, the anion-induced groups of −CF_3_, −SO_2_–CF_3_ and –NH–SO_2_–CF_3_ were calculated to be able to link with Te. Additionally, other groups such as − F (from the further reduction of − CF_3_) (*E*_ads_ =  − 0.93 eV) and − OH (from H_2_O) (*E*_ads_ =  − 0.67 eV) can also bond with Te. These simulation results are very good agreement with the above FTIR and XPS results.

Based on previous reports and our experimental findings, we propose the following possible delaminating mechanism for Te: (1) Upon applying an electric field, the anions and cations of the IL move to the corresponding electrodes. Their distribution may follow the Gouy–Chapman–Stern model, wherein cation–anion interactions (hydrogen–bond and electrostatic interactions) [79, 80], cation/anion–Te electrode adsorption interactions [81], and cation/anion–CH_3_CN hydrogen–bond interaction [82–84] need to be considered. Therefore, the possibility that both imidazolium cations and their counter NTf_2_ anions coexist on the Te or Pt electrodes cannot be overlooked [85]. (2) Specifically, taking into account the delamination current behavior illustrated in Fig. 1g, h, at + 5 V, Te is the cathode and Pt is the anode, and the imidazolium cations migrate to the Te electrode and, together with their reduction products, intercalate into Te crystals. Their large size and organic nature make the as-prepared nano-Te macroscopically and also microscopically large and flexible (probably forming cation-Te complexes) in CH_3_CN and provide faster Te stripping. Their steric bulk is also responsible for the difficulty in delaminating nano-Te with imidazolium-based products as compared with alkyl-side-chain-based radicals. At the Pt electrode, the anions aggregate and take place in multi-step electrochemical reactions, finally resulting in CHF_3_ [49]. The possible cation–Te complexes on the Te surface may hinder the reaction of nano-Te with the formed F-containing species, thus resulting in nano-Te with weak F signals (Fig. 2e). (3) In sharp contrast, at − 5 V, Te acts as the anode and Pt as the cathode. In this case, NTf_2_ anions accumulate near the Te and decomposition reactions occur, generating a series of active species that intercalate, strip, and chemically graft to Te. Interestingly, these inorganic species are smaller in both size and momentum, thus providing a slower rate of Te delamination and producing nano-Te in the form of small-sized fragments that float to the electrolyte surface. Possibly, because these species may have energy and volume matching with the surface of different-dimensional Te, the resulting nano-Te materials have multi-morphology and multi-dimensional high-entropy properties.

### Nano-Te-loaded PVA-based Foam Hydrogels

**Fig. 3:**
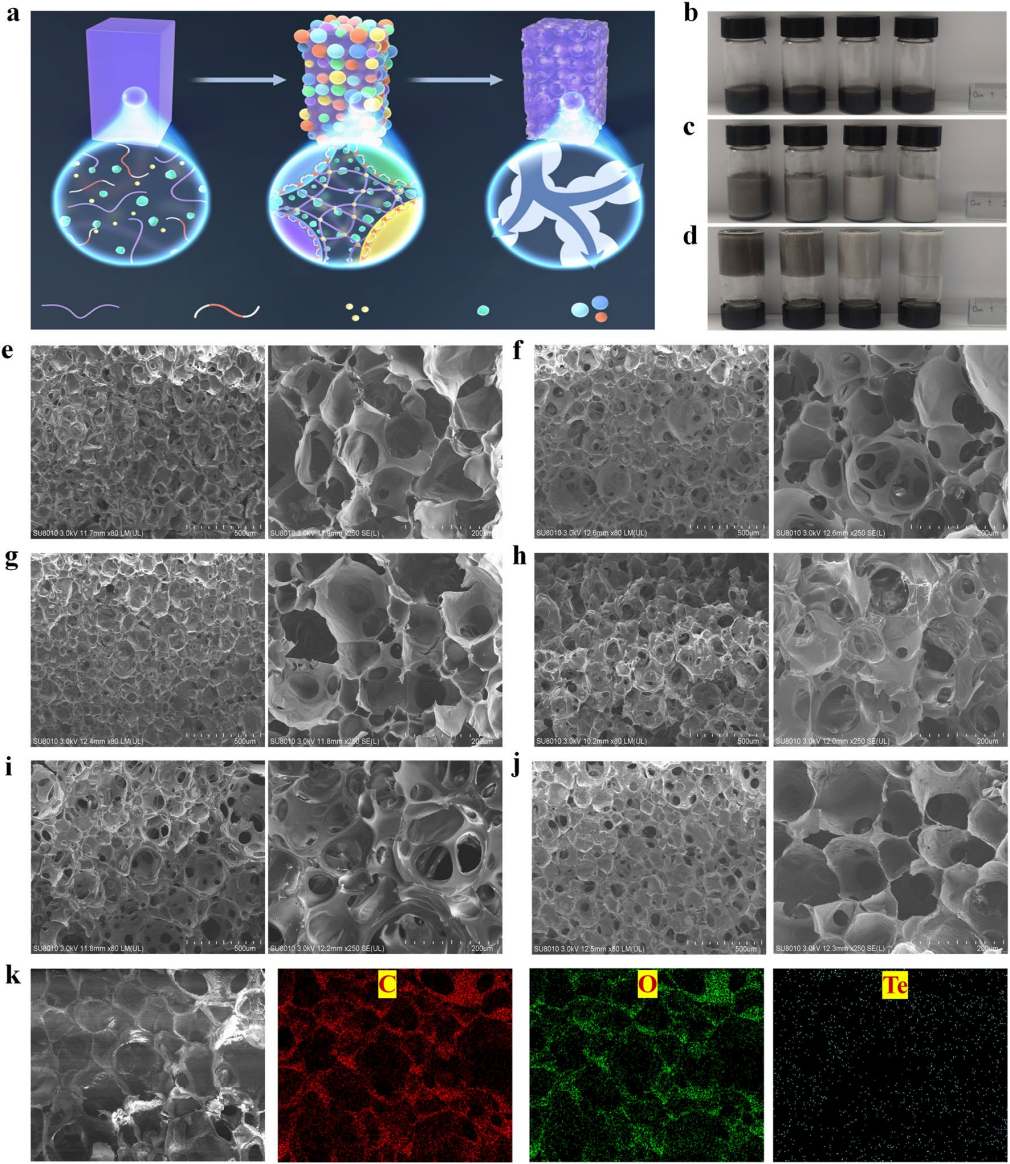
Construction of PVA/nano-Te-based foam hydrogels. **a** Schematic showing the formation of foam hydrogels, including homogeneous precursor solutions (PVA, F-127, nano-Te, GA, and HCl) (first step), foaming and cross-linked state (second step), and water-filled state (third step). Photographs of PVA/F-127/nano-Te foam hydrogels at the above corresponding stages, including **b** homogeneous precursor solution with various Te contents (from left to right: 0.37, 0.19, 0.09, and 0.05 wt%), **c** fully foaming state, and **d** heat-induced cross-linked state. SEM images showing the cross-sectional microstructures of **e** neat PVA foam hydrogel, **f** PVA/F-127 foam hydrogel, and **g–j** PVA/F-127/nano-Te foam hydrogels with nano-Te contents of **g** 0.05, **h** 0.09, **i** 0.19, and **j** 0.37 wt%. **k** Elemental mapping images of C, O, and Te for the 0.37 wt% Te sample

To realize the application of nano-Te to solar interfacial desalination, PVA-based foam hydrogels were explored as a model platform. As shown in Fig. 3a, by virtue of the excellent foaming ability of the PVA solution, we developed a foam phase inversion strategy to fabricate PVA/F-127/nano-Te foam hydrogels in which F-127, a triblock copolymer, acts as the foam stabilizer. We envisioned that these foam hydrogels would combine the performance characteristics of both foams and hydrogels. Briefly, the pre-mixed homogeneous PVA/F-127/nano-Te solutions were subjected to a high-speed rotation treatment (35,000 rpm, 180 s), generating a foam (Fig. S8). The addition of F-127 further homogenized and stabilized the foam. The foaming procedure was completed within 180 s and subsequent cross-linking between PVA and glutaraldehyde fully stabilized the hydrogel.

Figure 3b shows PVA/F-127/nano-Te precursor solutions having different nano-Te concentrations. Upon foaming (Fig. 3c), the volume of all the solutions increases at least twofold. Following heat treatment to initiate cross-linking, these volumes are effectively maintained (Fig. 3d). At this stage, the foam hydrogels are filled with air, so water is introduced to the pores by simple compression in water.

Typical cross-sectional morphologies of the PVA-based foam hydrogels are shown in Fig. 3e–j. Continuous and open pores are observed in the case of the PVA foam hydrogel without the addition of F-127 (Fig. 3e). Similar multi-porous structures are present in the PVA/F-127 foam hydrogels (Fig. 3f), indicating that these pores are mainly generated by the foaming conditions. Loading the PVA/F-127 foam hydrogel with nano-Te does not significantly affect its microstructure, regardless of the concentration of nano-Te (Fig. 3g–j). C, O, and Te elemental mapping images of the PVA/F-127/nano-Te sample (Fig. 3k) indicate the homogeneous dispersion of nano-Te within the foam hydrogels.

**Fig. 4:**
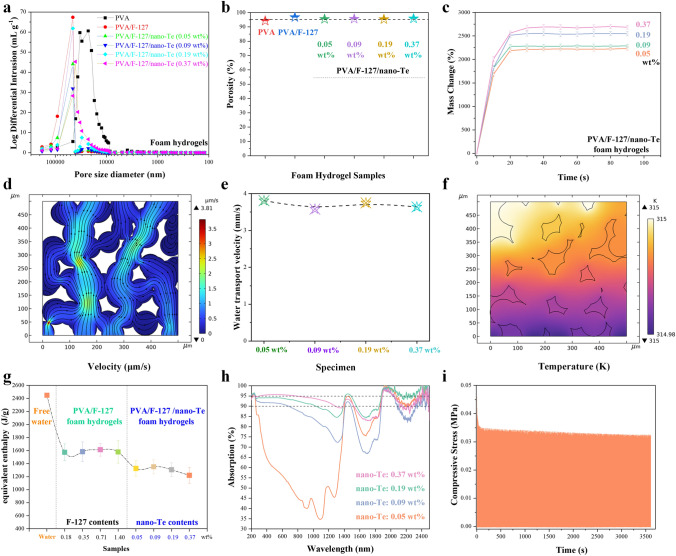
Combined features of the PVA/F-127/nano-Te foam hydrogels. Foam characteristics: **a** Pore size distribution by mercury intrusion method, **b** corresponding porosity, and **c** water uptake behaviors at room temperature. COMSOL models: **d** water and wet air transport behaviors by meshing effect, **e** corresponding Te content dependence of water transport velocity, and** f** heat energy distribution under 1 sun radiation. Hydrogel characteristics: **g** evaporation enthalpy of free water and water within the foam hydrogels. **h** Solar absorption spectra of seawater-wetted foam hydrogels with various Te contents. **i** Mechanical compressive stability of a sample with 0.37 wt% Te at a fixed compressive ratio of 85%

Polymeric foams are able to take up and rapidly transport large amounts water as well as exerting an anti-salting effect owing to their large open pores; however, this structure has no effects on the Δ*H*_E_ of water, which leads to low evaporation rates. Polymeric porous hydrogels can decrease Δ*H*_E_ and provide excellent water transport; however, these structures are limited by residual salt and/or dyes present in the water system when they are cleaned. Our PVA/F-127/nano-Te foam hydrogels have the aforementioned advantages of porous hydrogels while overcoming their limitations. As shown in Fig. 4a, the neat PVA foam hydrogel shows mainly large pores in the range of 10–100 μm with fewer smaller pores of several-micrometers diameter. Through the introduction of F-127 and both F-127 and nano-Te, the pores are enlarged. In addition, several pores in the range of 1–10 μm are also observed, indicating multiple pore sizes within these foam hydrogels, which is in good agreement with the TEM results. The foaming of PVA in foam hydrogels also results in its depression of crystallization (Fig. S9). The corresponding porosities of the PVA/F-127/nano-Te foam hydrogels are plotted in Fig. 4b. The PVA/F-127/nano-Te foam hydrogels show a high porosity of over 95%, similar to those of neat PVA foam hydrogel (94%) and the PVA/F-127 foam hydrogel (96.7%), respectively. The highly poly-porous structures of the foam hydrogels also result in fast water uptake, short equilibration time, and high water absorption (Fig. 4c). For example, a foam hydrogel with 0.37 wt% nano-Te can absorb ~ 2000 wt% water relative to its fully dried state within 10 s before saturating within another 10 s at a water uptake of 2750 wt%, as is consistent with typical foams. The results for all the samples are similar over the initial stage (0–10 s), where the water uptake velocity of the foam hydrogels is largely independent of the concentration of nano-Te.

The water transport and heat management within the porous foam hydrogels were also investigated by COMSOL simulations. By building meshing patterns, 2D transport velocity patterns of liquid water and wet air in the interconnected pores (overlapping circles in the COMSOL model) were generated (Fig. 4d). Capillary pressures drive water upward through linked pores, where it evaporates at the top surface under 1 sun solar energy. The water and vapor transport channel are almost a straight line owing to the high degree of pore interconnectivity, indicating negligible diffusion barriers. For the hydrogel samples with different Te loadings, the calculated total water and vapor transport velocities are shown in Fig. 4e, and they indicate that the addition of Te has little effect on the water transport properties of the PVA foam hydrogels. These results agree well with the water uptake velocity results shown in Fig. 4c.

Additionally, a simple heat-transfer model was used to describe the temperature distribution in the Te-0.05wt%-doped sample (Fig. 4f). The steady-state temperature distribution simulation predicted that the maximum temperature in the Te-0.05wt%-doped sample was 315 K at the surface of the polymeric network. This predicted value was close to our experimental value (see below), confirming the heat confinement effect of Te doping. The PVA-based foam hydrogels had a considerably lower value of Δ*H*_E_ for water evaporation, as shown in Fig. 4g. The experimental Δ*H*_E_ value (obtained by using a previously reported method) of approximately 1200 J g^−1^ observed in the case of the PVA/F-127/nano-Te foam hydrogel with 0.37 wt% nano-Te is half that of free water. The other PVA/F-127/nano-Te foam hydrogels also show decreased Δ*H*_E_ values that are superior to those of PVA/F-127 foam hydrogels without nano-Te. The highly porous structures of these foam hydrogels, particularly with the addition of nano-Te, which influences the pore distribution and effectively elevates the fraction of intermediate water, may be responsible for the decreased Δ*H*_E_ value [86].

Figure 4h shows the solar absorption behaviors of seawater-wetted PVA/F-127/nano-Te foam hydrogels, which strongly depend on nano-Te concentration. In addition, the sample with the highest nano-Te content absorbs light covering 95% of the UV–Vis region, 90% of the near IR region, and 85% of the far IR region, which may be attributed to the excellent solar absorption of the high-entropy nano-Te and the highly porous architecture of the foam hydrogel (Fig. S10). Additionally, the foam hydrogels are stable to compression, as shown in Figs. 4i and S11.

### Interfacial Evaporation Assessment

**Fig. 5:**
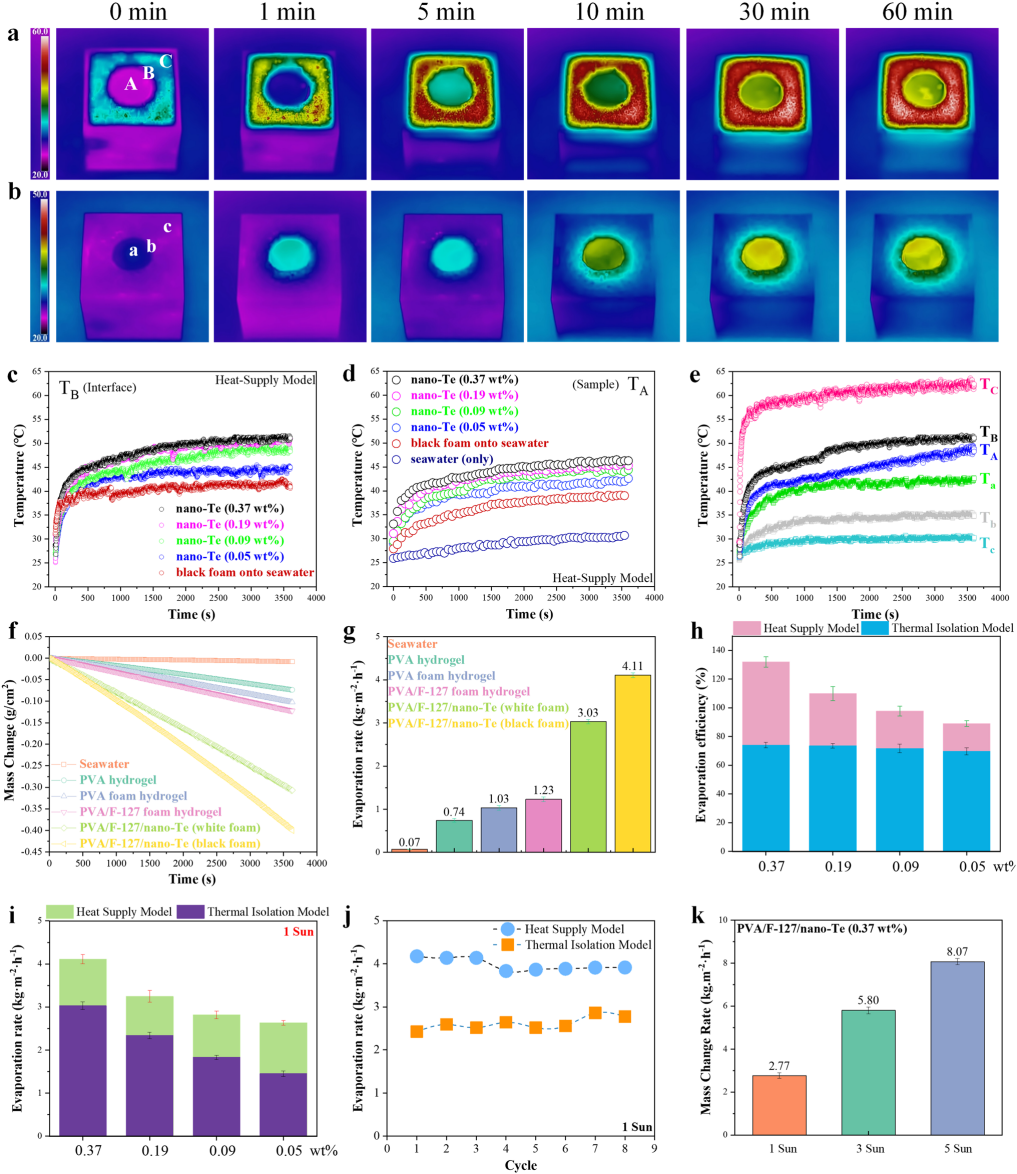
Solar desalination by foam hydrogels under 1 sun illumination indoors. **a** Thermal image of the heat-supply evaporation model, i.e., foam hydrogel (0.37 wt% Te) embedded into a commercial black foam. **b** Thermal isolation model generated by replacing black with white foam. **c–e** Temperature changes with time for sections A or a (sample central area), B or b (interface between sample and foam), and C or c (foam area distant from sample) for various samples in the above two models. **f** Plots of mass change over time for typical systems and **g** their corresponding evaporation rates (*ν*). **h, i** Energy efficiency (*η*) and *ν* of the PVA/F-127/nano-Te foam hydrogels with various Te concentrations by different models. **j** Evaporation stability of the 0.37 wt% Te sample over eight cycles. **k** Light intensity dependence of *ν* for the 0.37 wt% Te sample in the thermal isolation model

The PVA/F-127/nano-Te foam hydrogels were applied as solar evaporators embedded in a commercial black hydrophobic foam deployed as self-floating units on real seawater to examine their heat-supply evaporation behavior, as shown in Figs. 5a and S12. When irradiated under 1 sun, both the PVA/F-127/nano-Te foam hydrogel with 0.37 wt% Te and the surrounding black foam exhibit a gradual increase of temperature over time, where the black foam maintains a much higher temperature (Fig. 5a). Additionally, the interface area B between the sample and the black foam also shows higher temperatures than that of the central area A of the sample. The temperatures are directly proportional to the nano-Te concentrations of the samples, as shown in Fig. 5c, d. The black foam can absorb and transfer light into heat energy, which is partly absorbed by the sample, which has a relatively low temperature. This effect may be helpful for providing additional heating of interfacial water and eliminating in-plane heat loss between the sample and its surroundings. In the absence of the nano-Te samples, the black foam directly heats the central water compared with that without black foam (Fig. 5d). In contrast, when the black foam is replaced with white foam (Fig. S12), which is heat-insulative, the temperatures in areas a, b, and c are all lower than those for the heat-supply model with the same Te content, as shown in Fig. 5b, e. Higher interfacial temperatures are beneficial for desalination by solar evaporators. As shown in Fig. 5f, five typical PVA-based samples were tested for their evaporation capacity under 1 sun relative to bulk water. The samples used were the PVA hydrogel, PVA foam hydrogel, PVA/F-127 foam hydrogel, and the PVA/F-127/nano-Te with 0.37% Te in both heat-supply and heat-insulative models. For the heat-supply model, i.e., the PVA/F-127/nano-Te (black foam) case, the sample showed the best evaporation performance with an average evaporation rate (*ν*) of 4.11 kg m^−2^ h^−1^ (Fig. 5g) and an average energy efficiency (*η*) of 128% (Fig. 5h). The addition of F-127 further improves the evaporation rate of the PVA foam hydrogel, which may be due to the improved homogeneity of bubbles within the PVA foam system (Fig. 5f). Upon varying the Te content of the PVA/F-127/nano-Te samples, both the *ν* and *η* values change, with higher values derived from the heat-supply model (Fig. 5h, i). This excellent evaporation performance can be attributed to the evaporation system. First, the present foam hydrogels have lower ΔH_E_ and higher interfacial temperatures, the latter of which is largely relied on the heat-supply model that provided additional transferred heat energy to the evaporators, giving rise to a higher energy efficiency exceeding 100% [87]. Second, the system provides a hierarchical multiple pore structure that enables fast water transport and water supply.

The combination of these factors imparts the PVA/F-127/nano-Te foam hydrogels with superior performance compared with those of other typical solar evaporators in the literature (Table S1), such as interesting multi-functional fabrics [88–91] and three-dimensional (3D) hydrogels or xerogels [92–94]. Furthermore, our samples also exhibit long-term stability (Fig. 5j). Increasing light intensity further improves the evaporation performance for the thermal isolation model in Fig. 5k. However, this situation was not applicable to the heat-supply model because radiation over 3 suns can damage the black foam through excessive heating.

**Fig. 6:**
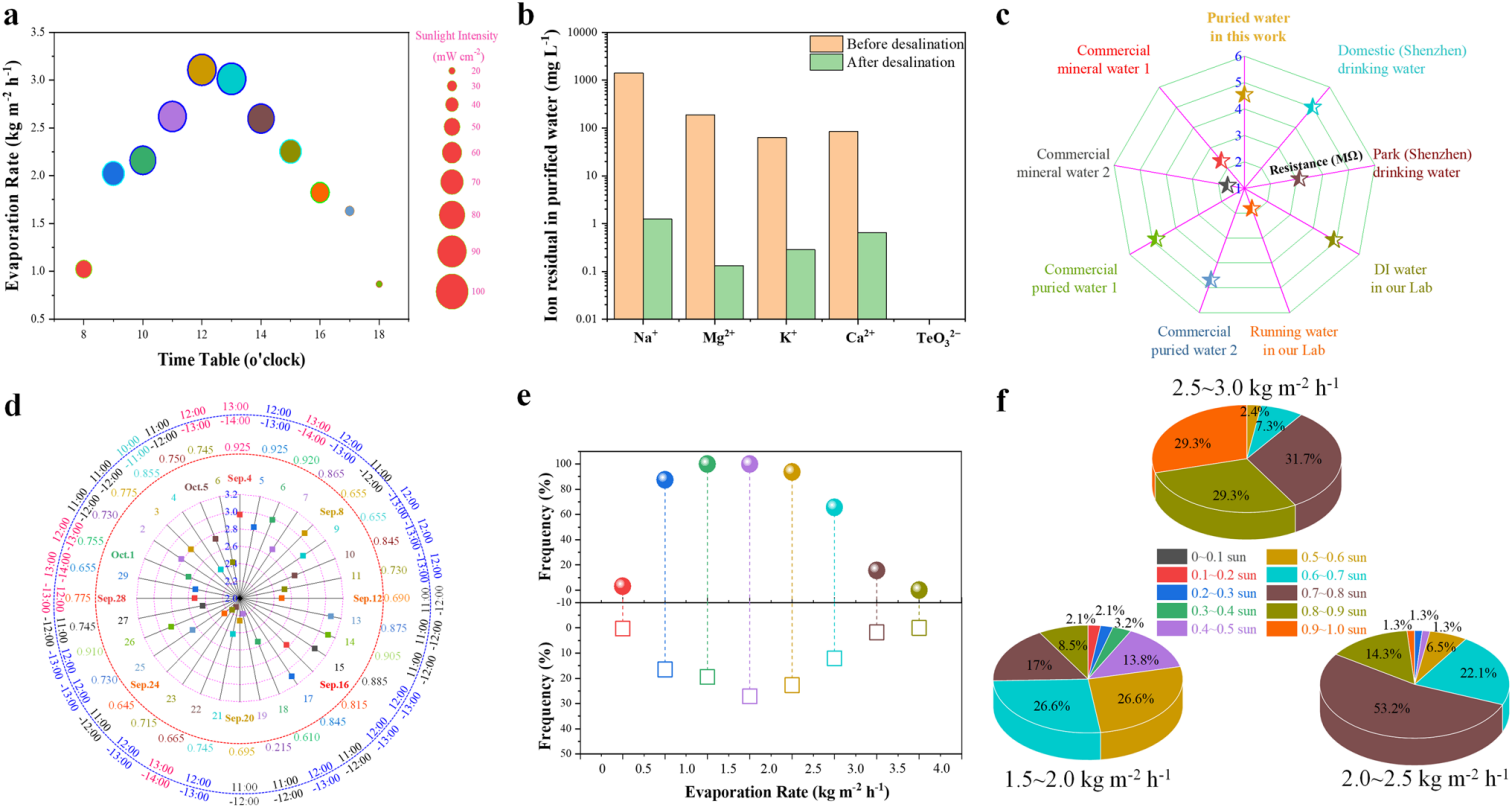
Solar desalination by the foam hydrogel with 0.37 wt% Te under natural light illumination in the heat-supply model outdoors during September 4 to October 6, 2022. **a** Real-time measurements of *ν* at different time points on September 14, 2022. Corresponding natural sunlight intensity was also recorded. **b** Ion concentration of real seawater and collected water before and after desalination.** c** Relative resistance of different types of water including seawater, collected water, drinking water, commercial pure water, and mineral water. **d** Maximum daily *ν* value measured during a 32-day testing period with the corresponding timetable and average light intensity relative to 1 sun. **e** Emergence of specific *ν* ranges during all days (top) and all the data points (down), including 0–0.5, 0.5–1.0, 1.0–1.5, 1.5–2.0, 2.0–2.5, 2.5–3.0, 3.0–3.5, and 3.5–4.0 kg m^−2^ h^−1^. **f** Corresponding light intensity region distribution, except for regions of 0–0.5, 0.5–1.0, 1.0–1.5, and 3.5–4.0 kg m^−2^ h^−1^

To explore the potential of the material for practical applications, we prepared a large-scale foam hydrogel and constructed a solar evaporator with the same proportions as those used above (Fig. S13). From September 4, 2022, to October 6, 2022, we monitored the evaporation rate, light intensity, ambient temperature, and central surface temperature of the system for 32 consecutive days of all-day monitoring (Fig. S14). For example, on September 14, 2022, as shown in Fig. 6a, the corresponding *ν* at different times correlates positively with the light intensity. On this day, the maximum value of *ν* was 3.11 kg m^−2^ h^−1^ between 11:00 and 12:00 with an average light intensity of 0.905 sun. The measured value of *ν* decreases at lower light intensity. Figure 6b shows the ion concentrations of the resultant collected water and seawater. After solar desalination, the concentrations of Na^+^, Mg^2+^, K^+^, and Ca^2+^ in the collected water decreased by three orders of magnitude compared with those before desalination. Moreover, no Te-based degradation products are detected, confirming the high purity and safety of the collected water. Furthermore, the solution resistances of several types of water were also measured to evaluate the quality of the collected water, as shown in Figs. 6c and S15. Our collected water shows a much higher resistance than tap water and park drinking water, and it is close to that of one of the commercially available purified water samples. In addition, the resistance of the collected water is only slightly lower than that of household drinking water, deionized laboratory water, and another type of commercially available pure water. Thus, the collected water is of high purity and may be drunk or sold directly.

To comprehensively evaluate the real evaporation performance of the sample outdoors, we thoroughly analyzed the 32-day test data and obtained the following results: During September 4 to October 6, the maximum values of *ν* are in the range 2.2–3.12 kg m^−2^ h^−1^, made up by 31%, 53%, and 16% of the time in the ranges 2.0–2.5, 2.5–3.0, and > 3.0 kg m^−2^ h^−1^, respectively. Thus, the evaporation rate is higher than 2.5 kg m^−2^ h^−1^ on approximately 70% of days (Fig. 6d). However, these maximum values are lower than those measured in the laboratory. A possible reason for this difference is that the maximum outdoor light does not provide the constant 1 sun energy input provided in the laboratory. For example, the maximum average light intensity within the ranges 0.8–0.9 and 0.9–0.95 sun accounts for only 22% and 13%, respectively, reflecting the instability of outdoor radiation. In addition, outdoor temperature and wind speed will affect the final evaporation rate. Evaporation rates in the ranges 0.5–1, 1–1.5, 1.5–2.0, 2.0–2.5, 2.5–3.0, and 3.0–4.0 kg m^−2^ h^−1^ accounted for 16.5%, 19.5%, 27.1%, 22.7%, 12.1%, and 1.7% of the 341 data points recorded, respectively; the corresponding days were 28 days (87.5%), 32 days (100%), 32 days (100%), 30 days (93.7%), 21 days (65.6%), and 5 days (15.6%), respectively (Fig. 6e). The light intensity coincides with the evaporation rate, as shown in Fig. 6f, and the sample maintains a relatively good evaporation rate even under weak sunlight. For example, the evaporation rate is within the range 2.5–3.0 kg m^−2^ h^−1^ (Fig. 6f) under high light intensity (0.8–1.0 sun), which accounted for approximately 60% of the data points, with the remaining 40% accounted for by the range of 0.5–0.8 sun. This value is even higher than the evaporation performance under the same conditions in the laboratory.

## Conclusions

The IL [C_3_NH_2_MIm][NTf_2_] can be electrolyzed into active species. The large imidazolium cation breaks down into imidazolium-based radicals that induce rapid swelling of bulk Te and generate an expanded porous structure. Conversely, the small [NTf_2_] anion of the IL degrades into smaller chemical species that induce fine exfoliation of bulk Te. The as-prepared nano-Te samples had 0D, 1D, and other morphologies, which are beneficial for absorbing solar light over a broad wavelength range at high intensity. The anion-induced nano-Te was terminated by F-, N-, and S-containing chemical groups, whereas only N- and S-containing groups were detected in the case of cation-induced nano-Te. Functionalization of Te can increase local disorder in its atomic arrangement, together with the multiple dimensions, such a strategy can greatly improve solar absorption and photothermal conversion, as revealed by our both experimental and simulation results.

In view of these findings, anion-induced nano-Te was integrated into PVA-based foam hydrogels to construct solar evaporators, which were evaluated under indoor and outdoor conditions. An evaporation rate of 4.11 kg m^−2^ h^−1^ was obtained with a corresponding energy efficiency of 128%. To the best of our knowledge, these are the highest values for semiconductor-based nanocomposites in the literature to date. In outdoor testing, under changing solar irradiation conditions, our solar evaporator exhibited evaporation rates in the range 2.5–3.0 kg m^−2^ h^−1^ under 0.5–1.0 sun. Thus, this study provides a new method for the preparation of functionalized nano-Te and an approach to developing high-performance water evaporation materials that function well under weak light radiation.

## Supplementary Information

Below is the link to the electronic supplementary material.

Supplementary file1 (39455 KB)

Supplementary file2 (40931 KB)

Supplementary file1 (PDF 1659 kb)
