# Supplementary material for: Chemical Scissors Tailored Nano-Tellurium with High-Entropy Morphology for Efficient Foam-Hydrogel-Based Solar Photothermal Evaporators
Source: Nanomicro Lett. 2023 Dec 8;16:47. doi: 10.1007/s40820-023-01242-y (PMC10709277; doi:10.1007/s40820-023-01242-y)
Supplement: Supplementary file 3 — Supplementary file1 (PDF 1659 kb) [file 40820_2023_1242_MOESM3_ESM.pdf]

Supporting Information for

Chemical Scissors Tailored Nano-Tellurium with High-Entropy Morphology for Efficient Foam-Hydrogel-Based Solar Photothermal Evaporators

Chenyang Xing[1, 2], Zihao Li[1, 2], Ziao Wang[3], Shaohui Zhang[5], Zhongjian, Xie[4], Xi Zhu[3, *] and Zhengchun Peng[1, 2, *]

[1] Key Laboratory of Optoelectronic Devices and Systems of Ministry of Education, Shenzhen University, Shenzhen 518060, P. R. China

[2] Center for Stretchable Electronics and NanoSensors, College of Physics and Optoelectronic Engineering, Shenzhen University, Shenzhen 518060, P. R. China

[3] School of Science and Engineering, The Chinese University of Hong Kong, Shenzhen, Shenzhen 518172, P.R. China

[4] Institute of Pediatrics, Shenzhen Children's Hospital, Shenzhen 518038, Guangdong, P. R. China

[5] International Collaborative Laboratory of 2D Materials for Optoelectronics Science and Technology of Ministry of Education, Institute of Microscale Optoelectronics, Shenzhen University, Shenzhen 518060, P. R. China

*Corresponding authors. E-mail: zhuxi@cuhk.edu.cn (Xi Zhu); zcpeng@szu.edu.cn (Zhengchun Peng)

Supplementary Figures and Table

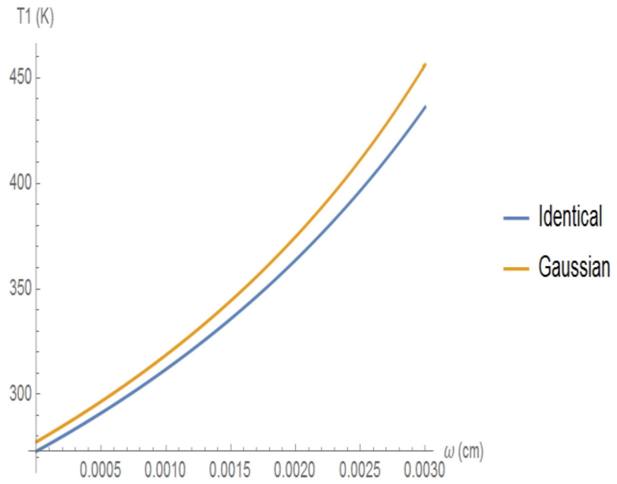

Fig. S1 Numerical results of $T_1^{identical}$ and $T_1^{Gaussian}$. The Gaussian distribution significantly elevated the temperature T_1

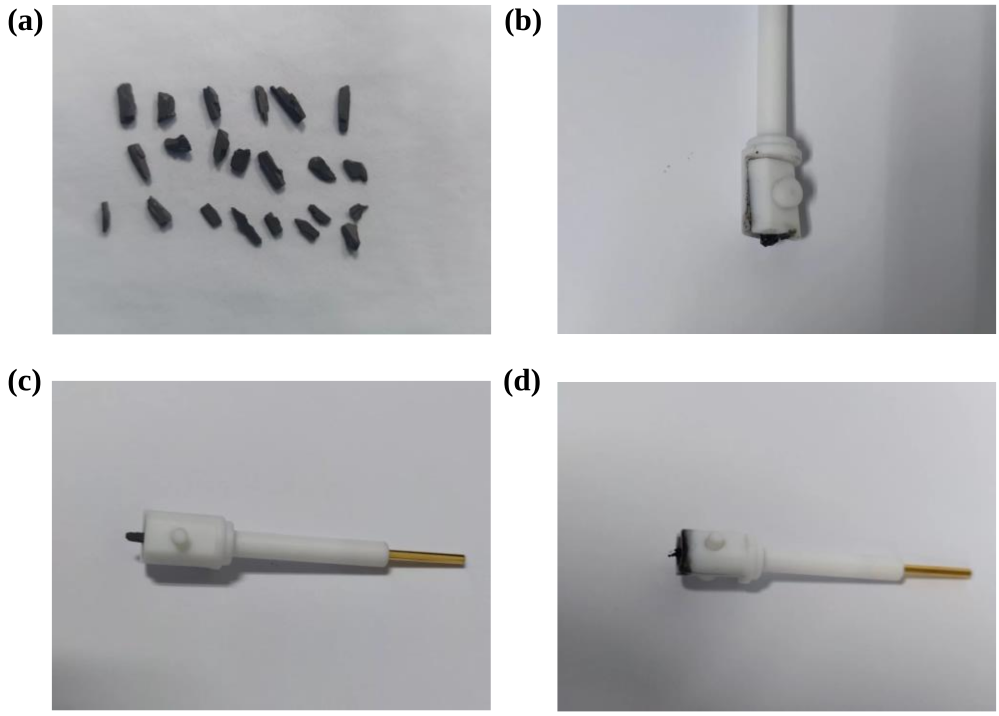

Fig. S2 (**a**) Commercial bulk Te. (**b**) Te working electrode after electrochemical reaction at + 5V condition. (**c**) and (**d**) Te working electrode before and after electrochemical reaction at - 5V condition

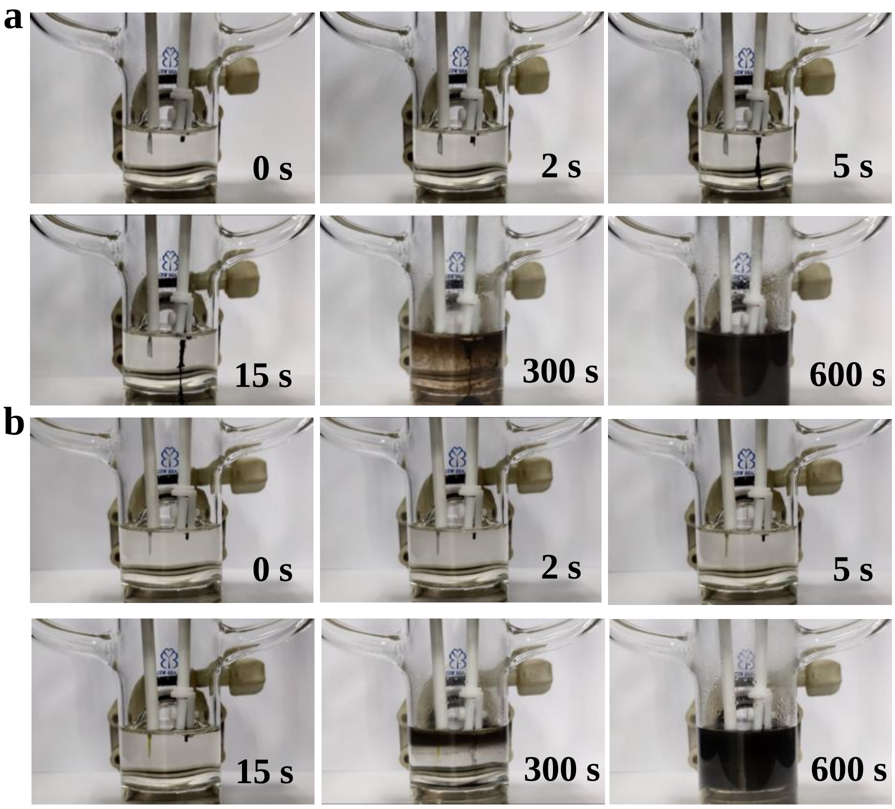

Fig. S3 Exfoliation process of Te at +5 V (**a**) and −5 V (**b**)

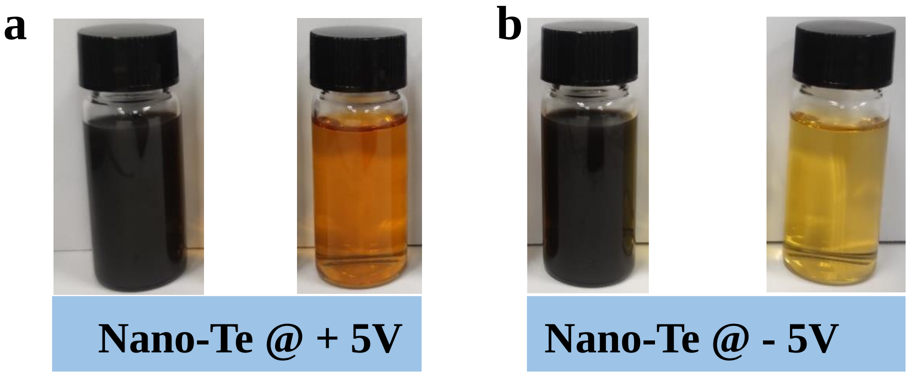

Fig. S4 As-prepared nano-Te dispersed in CH_3CN and corresponding filter liquors

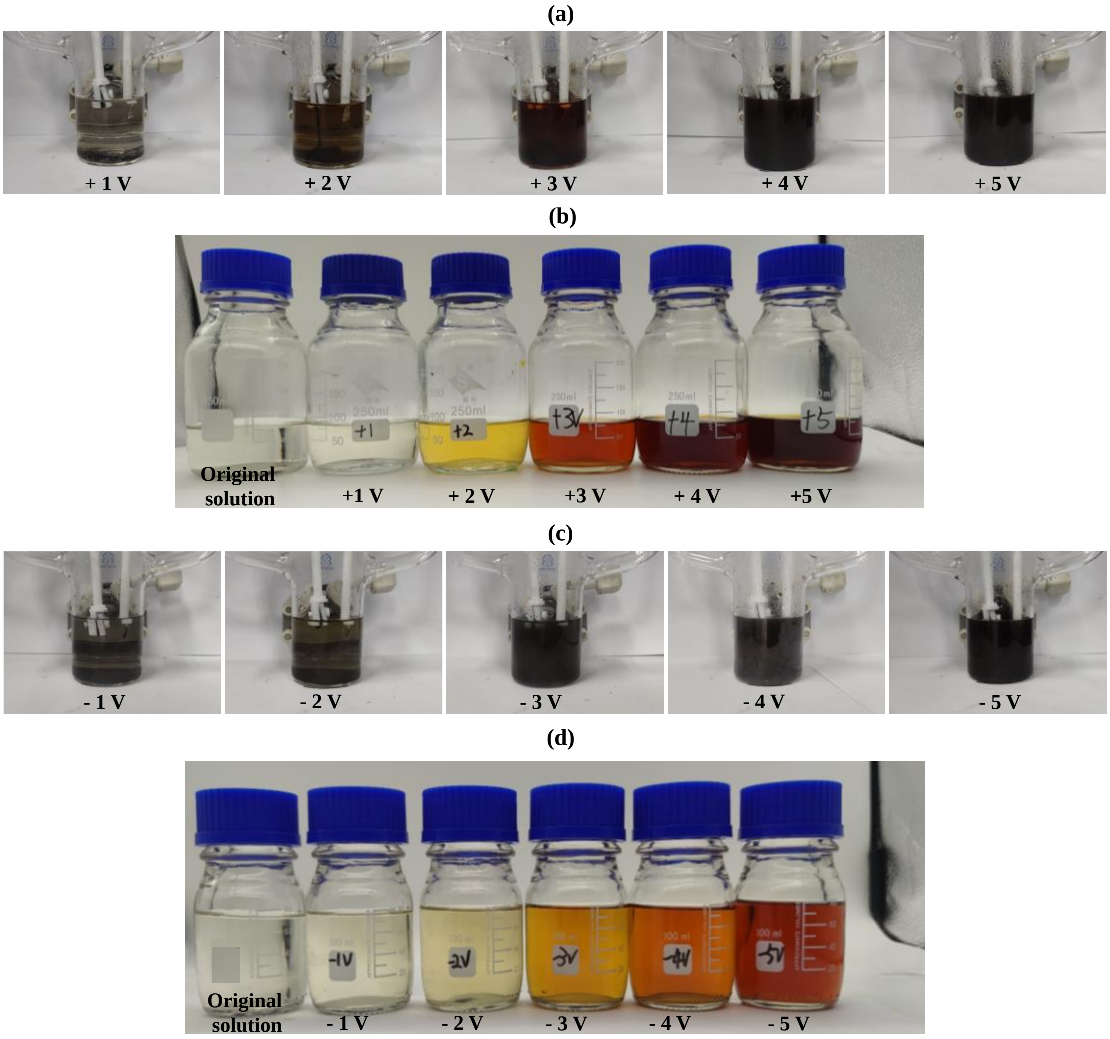

Fig. S5 (**a**) Photographs of Te exfoliation after 1 hour at different positive bias potentials (+1 V, +2 V, +3 V, +4 V and +5 V) in a three-electrode electrochemical reaction cell with CH_3CN as the electrolyte and a room temperature ionic liquid, $[C_3NH_2MIm][NTf_2]$ (0.125 M), as an intercalation agent. (**b**) the corresponding filter liquor after a vacuum-assisted filtration treatment. (**c**) and (**d**) similar photographs taken under different negative bias potential (–1, –2, –3, –4 and –5 V) conditions

At a low potential, such as +1, +2, –1 and –2 V, both cation and anion are relatively stable, especially the latter, and have not enough momentum to insert the chains of Te. And in this condition, the clear formation phenomenon of bubble cannot be observed. At a critical potential and higher, cation of IL has an improved kinetic energy that can realize the expanding and exfoliation of Te, and the anion of IL has also obtained energy to electrolyze and thus exfoliate Te. To this end, it is difficult to distinguish who (original ions or their electrolyzed products) exactly intercalated the Te's chains at early exfoliation stage.

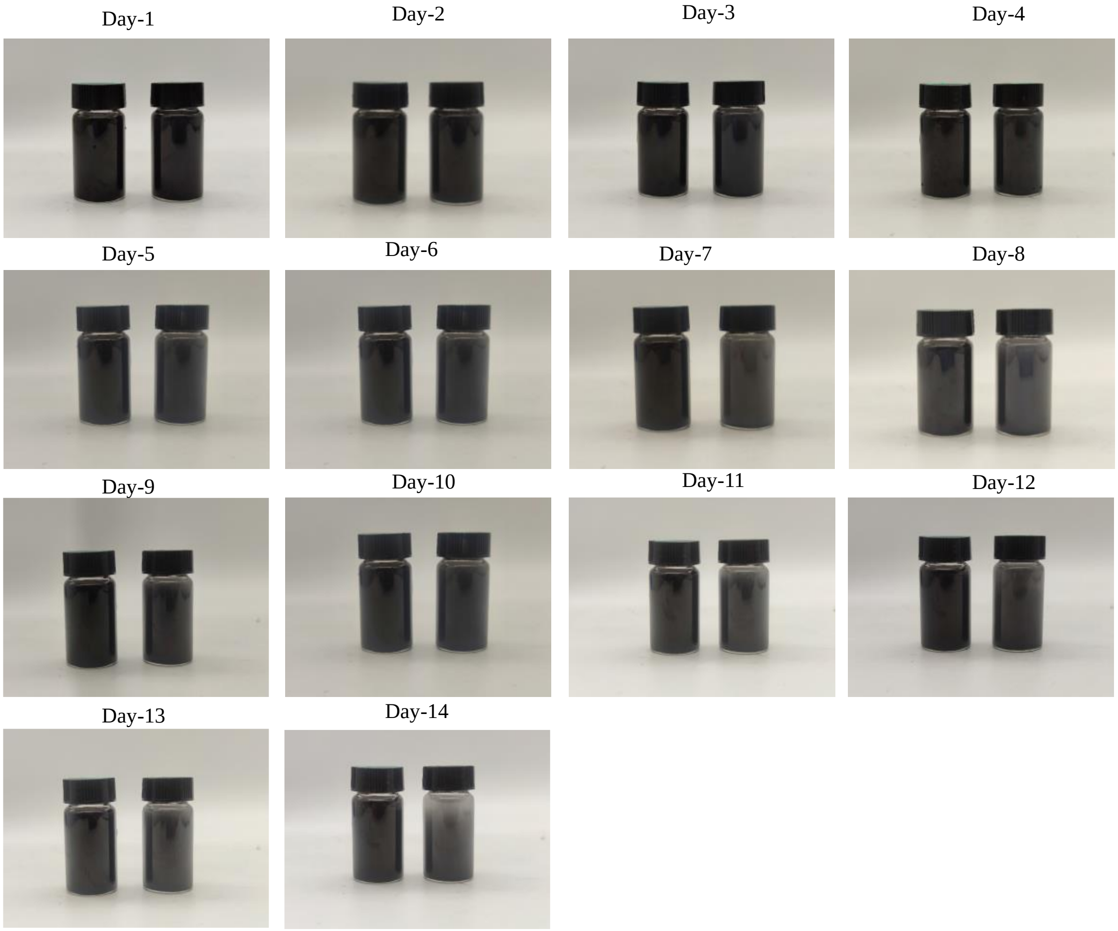

Fig. S6 Photographs of the colloidal solutions containing nano-Te/glutathione/H_2O (left) and nano-Te/H_2O (right) during 14-day observation at room temperature in air. The nano-Te powder was added into glutathione (GSH) solution (1.25 mg/mL) with a same weight ratio as GSH and the mixture was stirred at 500 rpm for 30 min

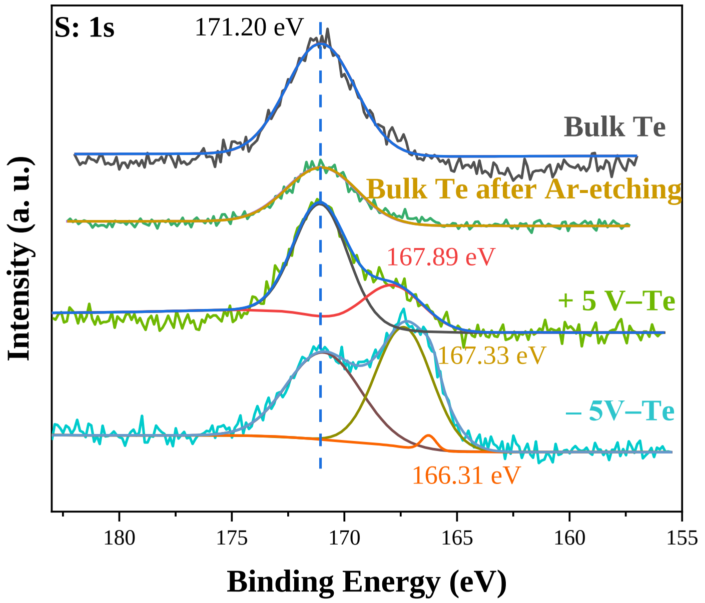

Fig. S7 XPS of samples for S element

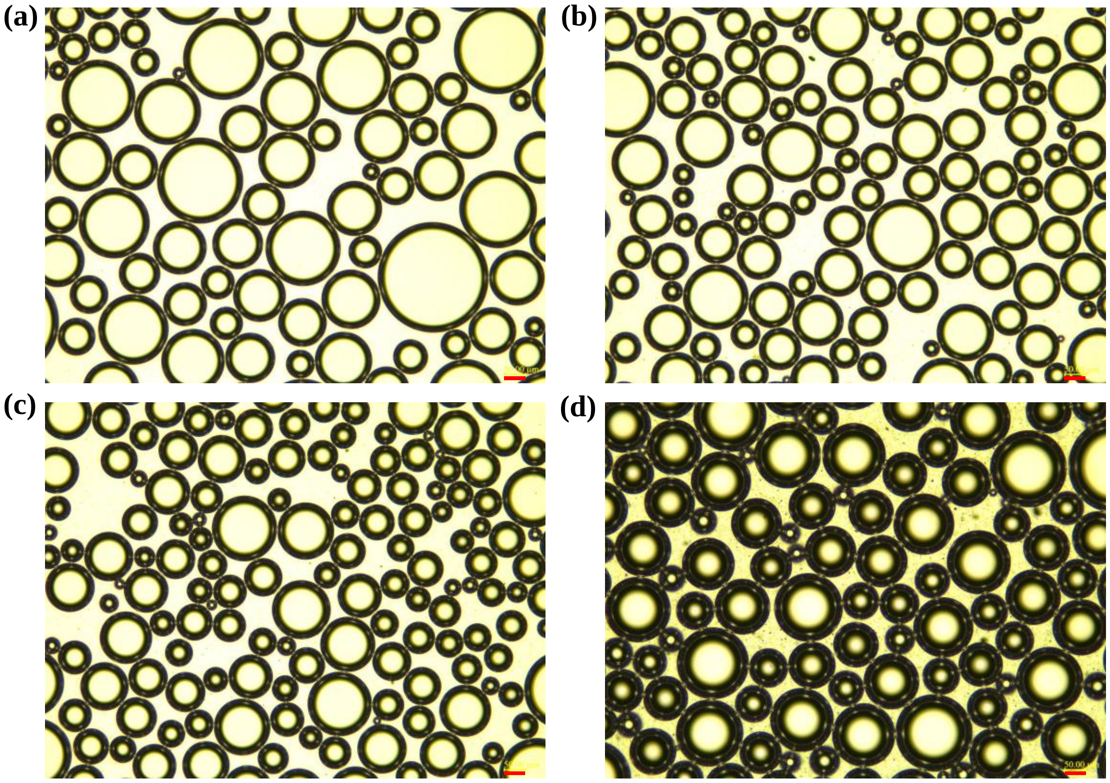

Fig. S8 Polarized optical microscope (POM) images of PVA-based foamy systems, wherein they contained PVA, F-127, nano-Te, GA, HCl and H_2O, respectively. The Te-concentrations in these systems are 0.05 wt% (**a**), 0.09 wt% (**b**), 0.19 wt% (**c**) and 0.37 wt% (**d**)

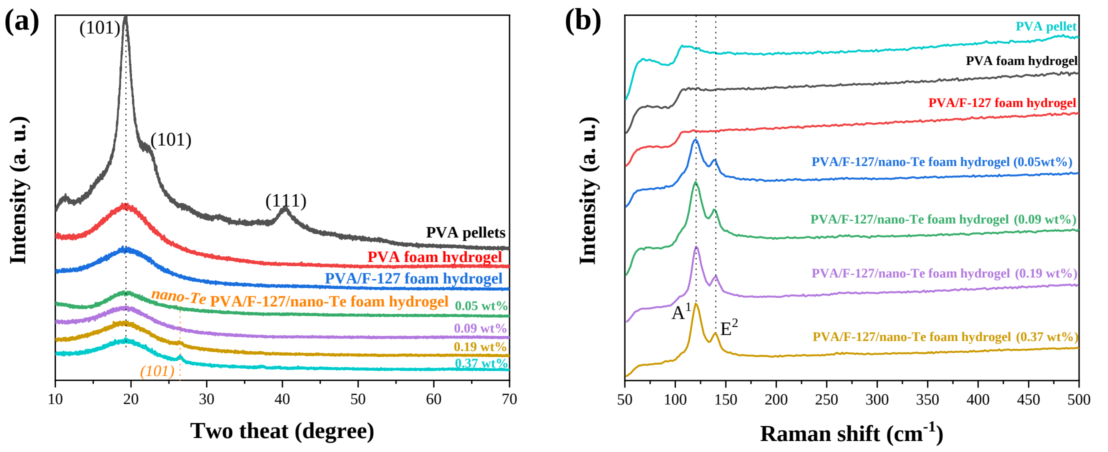

Fig. S9 (**a**) X-ray diffraction (XRD) patterns of samples including original PVA pellets, neat PVA foam hydrogel, PVA/F-127 foam hydrogel and PVA/F-127/nano-Te foam hydrogels with different Te concentrations (0.05, 0.09, 0.19 and 0.37 wt%). (**b**) Raman spectra of above samples, exhibiting the typical Te atom vibration behaviors in nano-Te component

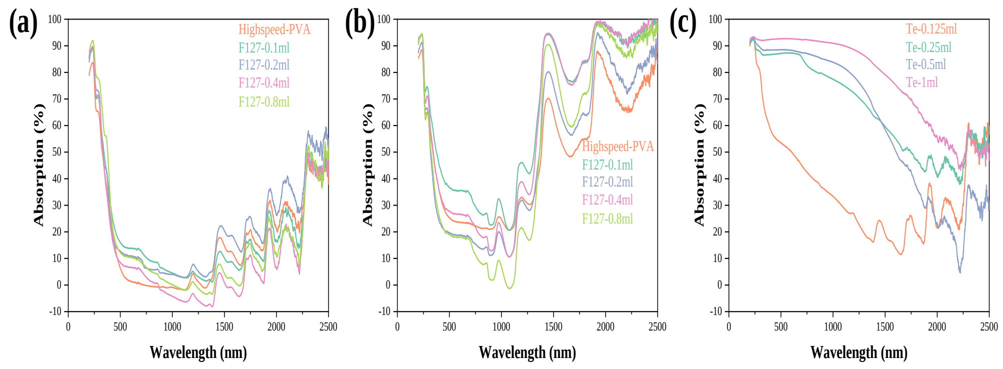

Fig. S10 Solar absorption behaviors of samples, including dried (**a**) and seawater-wet (**b**) PVA/F-127 foam hydrogels and dried PVA/F-127/nano-Te foam hydrogels (**c**)

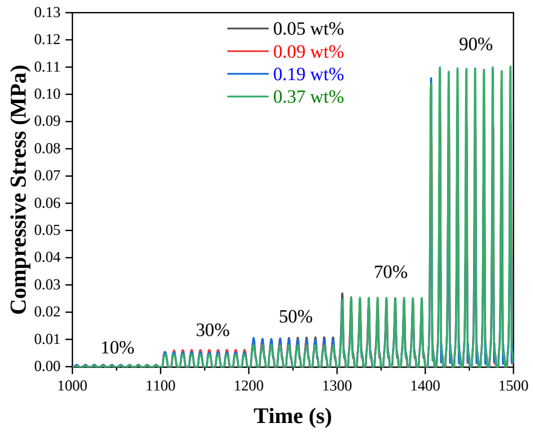

Fig. S11 Compressive property of PVA/F-127/nano-Te foam hydrogel with Te contents of 0.05, 0.09, 0.19 and 0.37 wt%, respectively, with various compressive ratios indicated above

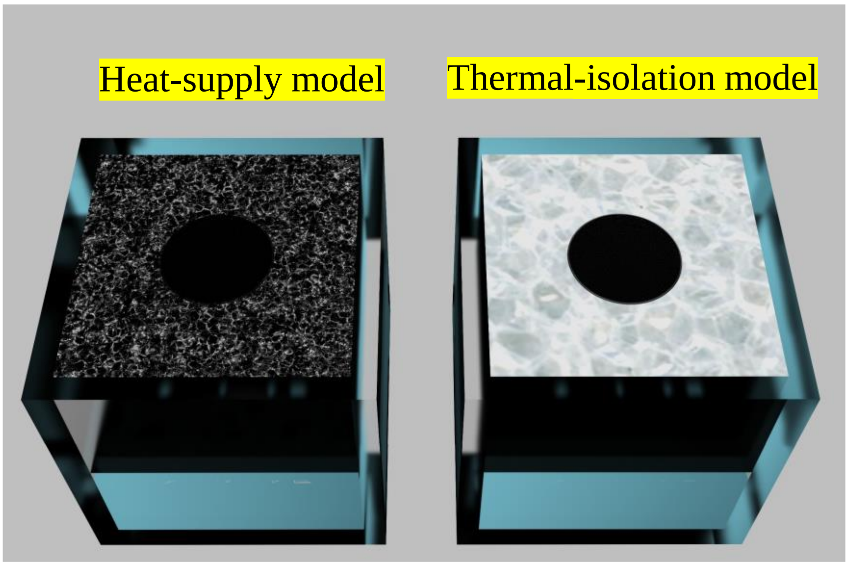

Fig. S12 Two contrasting evaporation models used in this work. The left was a heat-supply model, where PVA-based foam hydrogel was embedded within a commercial black foam. The right was a thermal-isolation model with a white foam replacing the black one. Typically, in the heat-supply model, the black foam had a higher photothermal induced temperature than that of foam hydrogel sample, generating a heat energy transfer from black foam to sample and conferring additional energy for sample. By contrast, in the thermal-isolation model, the sample had a higher temperature relative to the surrounding white foam and it lost small amount of energy into the white foam. As a result, foam hydrogel samples had higher temperatures in the heat-supply evaporation model to evaporate water.

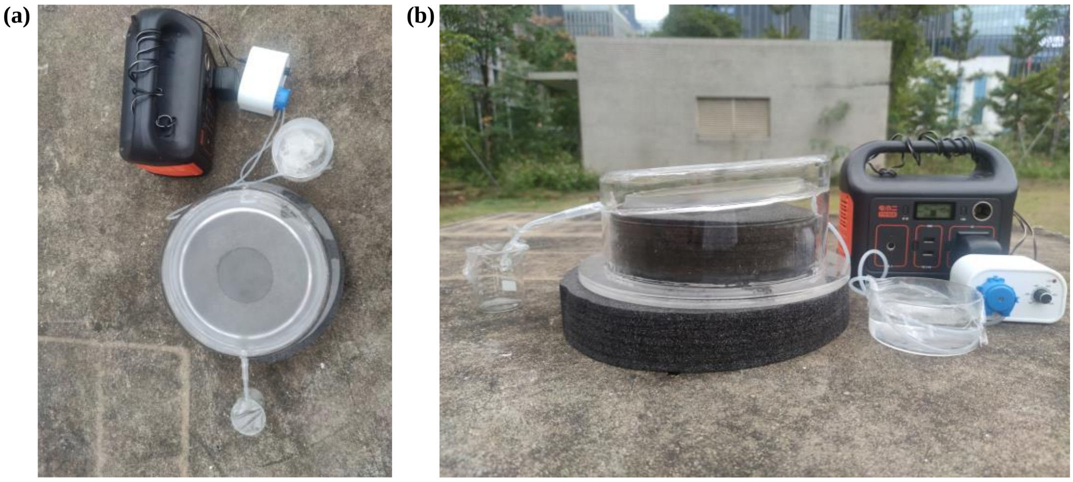

Fig. S13 Outdoor natural solar light desalination evaluation system, including a slant transparent quartz cover acting as moisture condenser, solar evaporator based on PVA/F-127/nano-Te (0.37 wt%) foam hydrogel and black foam with a heat-supply model, seawater supply equipment by a peristaltic pump under an external power supply. The collected water can be obtained through the water outlet designed at the sider of the condenser. (**a**) Top view and (**b**) side view of the system mentioned above

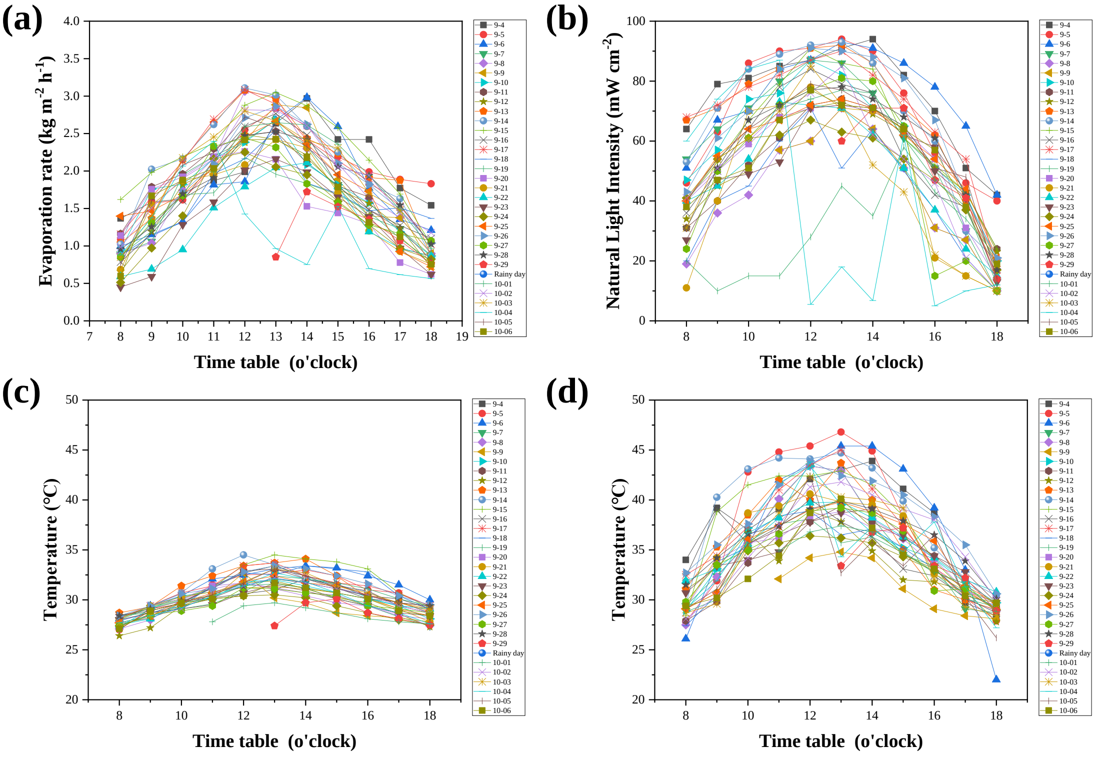

Fig. S14 Outdoor natural light desalination performance of PVA/-F-127/nano-Te (0.37wt%) foam hydrogel. The testing time period was from Sep. 4 to Oct. 6, 2022, except that the rainy day of Sep. 30, 2022. The respective real-time evaporation rate (**a**), light intensity (**b**), ambient temperature (**c**) and sample temperature at central position (**d**) were all carefully recorded

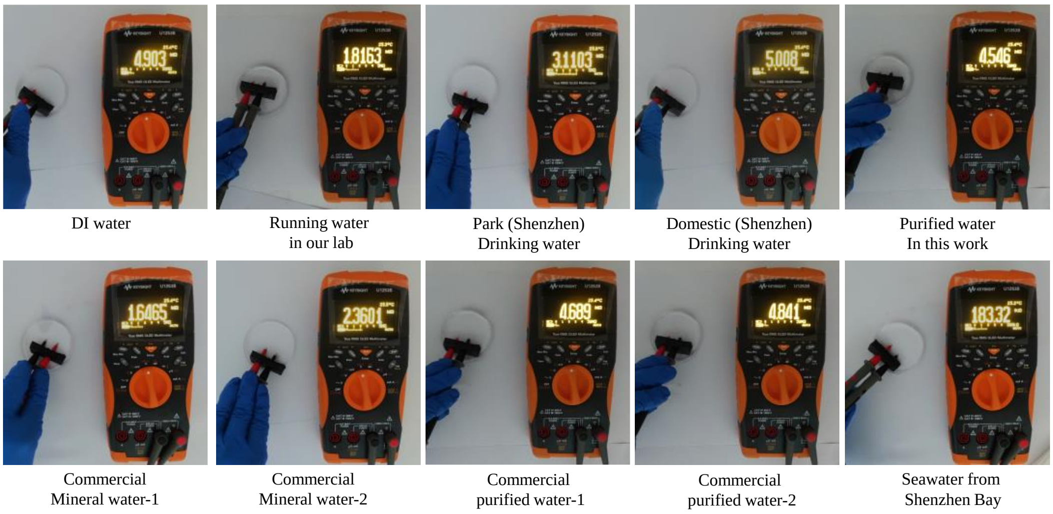

Fig. S15 Photographs showing resistance of various types of water, including deionized (DI) water and running water in our lab, direct drinking water from Dasha River Park and housing estate in Baishi Zhou of Shenzhen, China, purified water, commercial mineral and purified water and seawater from Shenzhen Bay, Shenzhen, China. During testing, the distance between two electrodes was fixed to insure the comparability of the reported resistance values of the above different kinds of water.

Table S1 The reported values of evaporation rate and energy efficiency of various photothermal evaporators under 1 sun illumination

Year	Solar system	Inorganic Material system	Evaporation Rate (1Sun) (kg m^{-2} h^{-1})	Energy efficiency (%)	*Ref.*
**2023**	**Foam Hydrogels**	**Te (0D/1D)**	**4.11**	**128%**	***This work***
2022	3D printed	GO + CB (2D/0D)	4.3	92	*(1) ACS Nano **16**, 2511-2520 (2022)*
2022	Hydrogel	Activated Carbon (0D)	4.14	94	*(2) Chem. Int. Ed. **61**, e202208487 (2022)*
2022	Aerogel	Graphene (2D)	4.11	None	*(3) Adv. Sci. **9**, 2205202 (2022)*
2022	3D fabric	MXene (2D)	3.95	177.8	*(4) Adv. Funct. Mater. **32**, 2205790 (2022)*
2019	Hydrogel	Ti_2O_3 (0D)	3.6	90	*(5) ACS Nano **13**, 7913-7919 (2019)*
2022	Hydrogel	CB (0D)	3.53	81.6	*(6) Adv. Mater. **34**, 2203137 (2022)*
2023	Hydrogel	CB (0D)	3.52	97.2	*(7) Chem. Eng. J. 458(141511 (2023).*
2023	xerogel	CB (0D)	3.39	95.6	*(8) Chem. Eng. J. **454**(140383 (2023).*
2022	Hydrogel	MoS_2 (2D)	3.297	93.4	*(9) Angew. Chem. Int. Ed. **61**, e202208587 (2022)*
2022	Hydrogel	rGO + Ag NPs (2D/1D)	3.20	95.67	*(10) Nano Energy **100**, 107441 (2022)*
2020	3D printing	CNT (1D)	2.63	96	*(11) Nat. Commun. **11**, 521 (2020).*
2018	3D foam	Graphene (2D)	2.6	87	*(12) ACS Nano **12**, 829-835 (2018)*
2022	Balsawood	(Fe+Co+Ni+Ti+V+Cr +Mn+Cu) High-entropy-alloy (0D)	2.58	None	*(13) Adv. Energy Mater. **12**, 2203057 (2022)*
2020	Membrane	MoS_{2-x} (2D)	2.50	89.6	*(14) Adv. Mater. **32**, 2001544 (2020)*
2022	Membrane	CNT (1D)	2.46	91.14	*(15) Adv. Funct. Mater. **32**, 2113264 (2022)*
2021	Fabric	CuS (1D)	2.27	90.2	*(16) ACS Nano **15**, 13007-13018 (2021)*
2023	MOF	Co^{2+}	2.2	91.1	*(17) Appl Catal. B. **337**(123001 (2023).*
2022	Film	Porous Carbon (3D)	2.12	112.8%	*(18) Sol. RRL 2200803 (2022).*
2022	Hydrogel	Carbon Fiber (1D)	2.13	94.2	*(19) EcoMat **5**, e12282 (2022)*
2022	Aerogel	Carbon (0D)	1.89	85	*(20) EcoMat **4**, e12216 (2022)*
2020	Wood	Fe (0D)	1.8	None	*(21) Nano Energy **74**, 104886 (2020)*
2018	Aerogel	Graphene (2D)	1.78	91	*(22) Nano Energy **46**, 415-422 (2018)*
2020	Porous Structure	La0.7Sr0.3CoO_3 (0D)	1.67	92	*(23) Nano Energy **70**, 104538 (2020)*
2019	Aerogel	CuS (3D)	1.63	94.9	*(24) Nano Energy **56**, 708-715 (2019)*
2020	Cloth	$Zn_1Cu_{0.2}$-MOF(3D)	1.63	91	*(25) Energy Environ. Sci. **13**, 4891-4902 (2020)*
2016	Aerogel	GO+CNT (2D/1D)	1.622	83	*(26) Adv. Mater. **29**, 1604031 (2017)*
2018	Polystyrene Foam	CB (0D)	1.59	96	*(27) Joule **2**, 1331-1338 (2018)*
2022	Membrane	Fe (0D)	1.539	90.2	*(28) Chem. Mater. **34**, 10399-10408 (2022).*
2021	Membrane	MXene (2D)	1.53	85.6	*(29) ACS Appl. Nano Mater. **4**, 14274-14284 (2021)*
2022	Film	$Cu_{99}Au_1$ (0D)	1.51	94.5	*(30) Adv. Mater. **34**, 2200108 (2022)*
2019	Film	Cu-CAT-1 MOF (3D)	1.5	96	*(31) Adv. Mater. **31**, 1808249 (2019)*
2018	Aerogel	CNT(1D)	1.4406	86.8	*(32) Adv. Energy Mater. **9**, 1802158 (2019)*
2020	Porous Structure	CNT (1D)	1.41	95.8	*(33) Nano Energy **74**, 104875 (2020)*
2021	Cotton Fabric	MnO_2 (2D)	1.4	87.48	*(34) ACS Appl. Nano Mater. **4**, 13724-13733 (2021)*
2017	Foam	Graphene (2D)	1.4	93.4	*(35) Adv. Mater. **29**, 1702590 (2017)*
2021	Membrane	Bi_2O_3 (1D)	1.38	91.1	*(36) Adv. Funct. Mater. **31**, 2100703 (2021)*
2022	Janus Fabric	CB (0D)	1.37	91.3	*(37) Adv. Funct. Mater. **32**, 2113258 (2022)*
2021	Sponge	CNT (1D)	1.34	>90	*(38) J. Mater. Chem. A **9**, 17502-17511 (2021)*
2019	Foam	Cu + Al_2O_3+CB (3D/2D/0D)	1.31	79.8	*(39) Sci. Adv. **5**, eaaw7013 (2019)*

2018	Janus Membrane	CB (0D)	1.3	72	*(40) Adv. Energy Mater.* ***8****, 1702884 (2018)*
2018	Wood	Graphite	1.15	80	*(41) Adv. Funct. Mater.* ***28****, 1707134 (2018)*
2020	Membrane	TiO_2@(Ag) (0D/0D)	0.82	82 (6sun)	*(42) ACS Appl. Nano Mater.* ***3****, 10895-10904 (2020)*
2016	Membrane	Al (0D)	5.7 (4sun)	90	*(43) Nat. Photon.* ***10****, 393-398 (2016)*
2022	Membrane	Graphene (2D)	6.72 kg $m^{-2}h^{-1}$	None	*(44) Adv. Mater.* ***34****, 2109718 (2022)*
2022	MOF-derive	Nanoporous Carbon(1D)	0.195 L $kg_{carbon}^{-1}\ h^{-1}$	None	*(45) Nat. Nanotechnol.* ***17****, 857-863 (2022)*
**Year**	**Solar system**	**Organic or Organic-Inorganic Material system**	**Evaporation Rate(1Sun) (kg m^{-2} h^{-1})**	**Energy efficiency (%)**	***Ref.***
2020	Hydrogel	OTS	4.0	93	*(46) Energy Environ. Sci.* ***13****, 2087-2095 (2020)*
2019	Hydrogel	Polypyrrole	3.6	92	*(47) Sci. Adv.* ***5****, eaaw5484 (2019)*
2021	3D structure	TPA-BTDH	3.6	None	*(48) Adv. Mater.* ***33****, 2102258 (2021)*
2022	Hydrogel	Modified needle coke	3.18	99	*(49) Adv. Mater.2207262 (2022).*
2021	PS sphere	Polypyrrole	2.6	None	*(50) Adv. Funct. Mater.* ***31****, 2102618 (2021)*
2019	Paper	Polypyrrole	2.12	91.5	*(51) Adv. Mater.* ***31****, 1900720 (2019)*
2019	Bilayer Foam	Polypyrrole	1.57	90.4	*(52) Nano Energy* ***60****, 841-849 (2019)*
2022	Plastic MGP Hybrid	PANI + GO (2D) + MXene (2D)	3.94	135.6	*(53) Adv. Funct. Mater.* ***32****, 2110636 (2022)*
2022	Wood	Zeolitic imidazolate framework-8	2.70	86	*(54) Nano Energy* ***95****, 107016 (2022)*
2022	PPy@MNF	Fabric	2.61	None	*(55) Sci. China Mater. 65(9), 2479-2490 (2022).*
2022	Fabric	Polypyrrole +ATP+Al (0D)	2.23	97.3	*(56) ACS Appl. Energy Mater.* ***5****, 13031-13041 (2022)*
2023	Fabric	PANI + CNT	2.06	None	*(57) Small. 2303716 (2023).*
2022	Fabric	PDA+CB	1.68	91.5	*(58) Sep. Purif. Technol. 278(119621 (2021).*
2021	Fabric	PPy+CNT	1.61	91.2	*(59) ACS Appl Mater Interfaces. 13(21), 24945-24956 (2021).*
2021	Membrane	$NiCo_xS_y$ (2D) + PANI	1.30	78.7	*(60) ACS Appl. Energy Mater.* ***4****, 3563-3572 (2021)*
2020	Flim	TiO_2(0D) and Polypyrrole	2.9(2sun)	97.3	*(61) Nanoscale* ***12****, 9680-9687 (2020)*
2021	Luffa Sponge	Carbonized lufa sponge	3.7	None	*(62) Sci. Rep.* ***11****, 16811 (2021).*
2020	Wood	Carbonization Wood	2.2	87	*(63) Nano Energy* ***78****, 105322 (2020)*
2020	Wood	Carbonization Balsa Wood	1.35	91.5	*(64) Energy Environ. Sci.* ***15****, 5405-5414(2022)*
2021	wood	Carbonization Wood	1.35	82	*(65) Energy Environ. Sci.* ***14****, 5347-5357 (2021)*
2019	Wood	Carbonization Balsa Wood	0.80	57	*(66) Energy Environ. Sci.* ***12****, 1558-1567 (2019)*
2022	Polystyrene Foam	Black paint	1.27	91	*(67) Nat. Commun.* ***13****, 849 (2022)*

GO: graphene oxide; CB: Carbon black; rGO: reduced graphene oxide; CNT: Carbon nanotube; MOF: Metal Organic Framework; CAT-1: Catecholates; OTS: Trichloro(octadecyl)silane; TPA-BTDH: a typical D-A-D molecule; ATP: attapulgite; polydopamine (PDA); PANI: Polyaniline
